# Two genes, ANS and UFGT2, from Vaccinium spp. are key steps for modulating anthocyanin production

**DOI:** 10.3389/fpls.2023.1082246

**Published:** 2023-02-02

**Authors:** Han M. Nguyen, Joanna Putterill, Andrew P. Dare, Blue J. Plunkett, Janine Cooney, Yongyan Peng, Edwige J. F. Souleyre, Nick W. Albert, Richard V. Espley, Catrin S. Günther

**Affiliations:** ^1^ The New Zealand Institute for Plant and Food Research Ltd, Auckland, New Zealand; ^2^ University of Auckland, School of Biological Sciences, Auckland, New Zealand; ^3^ The New Zealand Institute for Plant and Food Research Ltd, Hamilton, New Zealand; ^4^ The New Zealand Institute for Plant and Food Research Limited, Palmerston North, New Zealand

**Keywords:** anthocyanins, chalcone synthase, anthocyanidin synthase, UDP-glucose:flavonoid 3-O-glucosyltransferase, blueberry, bilberry, vaccinium

## Abstract

Anthocyanins are a major group of red to blue spectrum plant pigments with many consumer health benefits. Anthocyanins are derived from the flavonoid pathway and diversified by glycosylation and methylation, involving the concerted action of specific enzymes. Blueberry and bilberry (*Vaccinium* spp.) are regarded as ‘superfruits’ owing to their high content of flavonoids, especially anthocyanins. While ripening-related anthocyanin production in bilberry (*V. myrtillus*) and blueberry (*V. corymbosum*) is regulated by the transcriptional activator MYBA1, the role of specific structural genes in determining the concentration and composition of anthocyanins has not been functionally elucidated. We isolated three candidate genes, *CHALCONE SYNTHASE* (*VmCHS1*), *ANTHOCYANIDIN SYNTHASE* (*VmANS*) and *UDP-GLUCOSE : FLAVONOID-3-O-GLYCOSYLTRANSFERASE* (*VcUFGT2*), from *Vaccinium*, which were predominantly expressed in pigmented fruit skin tissue and showed high homology between bilberry and blueberry. *Agrobacterium*-mediated transient expression of *Nicotiana benthamiana* showed that overexpression of *VcMYBA1* in combination with *VmANS* significantly increased anthocyanin concentration (3-fold). Overexpression of *VmCHS1* showed no effect above that induced by *VcMYBA1*, while *VcUFGT2* modulated anthocyanin composition to produce delphinidin-3-galactosylrhamnoside, not naturally produced in tobacco. In strawberry (*Fragaria × ananassa*), combined transient overexpression of *VcUFGT2* with a *FLAVONOID 3*´*,5´-HYDROXYLASE* from kiwifruit (*Actinidia melanandra*) modulated the anthocyanin profile to include galactosides and arabinosides of delphinidin and cyanidin, major anthocyanins in blueberry and bilberry. These findings provide insight into the role of the final steps of biosynthesis in modulating anthocyanin production in *Vaccinium* and may contribute to the targeted breeding of new cultivars with improved nutritional properties.

## Introduction

Anthocyanins are a diverse group of natural plant pigments, providing a full spectrum of visible colours ranging from bright orange, pink and red to purple and blue (Chen, 2015). In plants, the colours provided by anthocyanins are known for their roles in attracting pollinators and seed dispersers, especially in reproductive organs such as flowers and fruits (Jimenez-Garcia et al., 2013). In many plant species anthocyanins only accumulate during fruit ripening, particularly in fruit skin, which makes these pigments visual cues for ripeness (Jimenez-Garcia et al., 2013).

As polyphenolic pigments, anthocyanins form part of the flavonoid phytochemical group and also act as antioxidants, photoprotectants, and metal-chelating agents (Stintzing and Carle, 2004; Landi et al., 2015). Anthocyanins are composite molecules, consisting of an anthocyanidin moiety which is linked with a glycoside at the C3-position of the heterocyclic C-ring structure (Castañeda-Ovando et al., 2009).

There are over 600 anthocyanins identified in nature, diversified primarily by different glycosylation patterns and other chemical modifications such as methylations and acylations (Smeriglio et al., 2016). Based on hydroxylation of the B-ring, there are three main groups of anthocyanidins: pelargonidin, cyanidin and delphinidin. Subsequent methylation of cyanidin generates peonidin, while methylation delphinidin produces petunidin and malvidin (Castañeda-Ovando et al., 2009).

The regulation of anthocyanin production in plants is highly conserved (Zhang et al., 2014). Activation of anthocyanin biosynthesis is transcriptionally controlled by the MBW complex, which is formed by subgroup 6 of the R2R3-MYB (R2R3-MYB) transcription factor (TF) family, the bHLH TF family and the WD Repeat (WDR) proteins (Zhang et al., 2014). To regulate the production of anthocyanins, R2R3-MYB TFs directly interact with promoters of structural genes from the anthocyanin biosynthesis pathway through their highly conserved DNA binding domain. It is the overall activity of the R2R3-MYB TFs that is thought to be the main determinant for the amount of anthocyanin produced (Schwinn et al., 2006; Allan et al., 2008). Furthermore, meta-analysis shows that overexpression of MYB TFs significantly impact the regulation of a set of structural genes together with increased anthocyanin production (Liu et al., 2021b). However, the manipulation of wide-acting MYB TFs can potentially lead to collateral consequences. For example, a red-fleshed transgenic apple line with high anthocyanin accumulation in fruit flesh was generated by constitutive expression of *MdMYB10* (Espley et al., 2013), but early browning of fruit flesh was commonly observed (Espley et al., 2019). Therefore, altering the expression of key structural genes of the flavonoid pathway is an alternative strategy to provide more specific targets for modulating anthocyanin production in fruits.

The anthocyanin biosynthesis pathway in plants has been well researched across different species (Liu et al., 2021a) and can be subdivided into three sections: the general phenylpropanoid pathway, the flavonoid pathway and the specific biosynthesis of anthocyanins (Zhang et al., 2014). The general phenylpropanoid pathway first utilises phenylalanine to synthesise *p*-coumaroyl CoA. Chalcone synthase (CHS) then directs the carbon flux into the flavonoid pathway *via* the conversion of *p*-coumaroyl CoA into naringenin chalcone. Subsequent activities of chalcone isomerase (CHI) and flavanone 3-hydroxylase (F3H) then produce the structural flavonoid backbone, which is further modified and converted to leucoanthocyanidins by dihydroflavonol 4-reductase (DFR). As part of the anthocyanin-specific biosynthesis pathway, anthocyanidin synthase (ANS) converts leucoanthocyanidins into coloured anthocyanidins, which are then stabilised by esterification with sugar molecules by glycosyltransferases such as UDP-glucose:flavonoid-3-*O*-glycosyltransferase (UFGT) (Liu et al., 2018). The positive relationship between anthocyanin accumulation and the expression of structural genes in the pathway has been documented in various plant species. For example, significantly reduced anthocyanin production was found in kiwifruit when *CHS* or *UFGT* expression were silenced using RNAi (Montefiori et al., 2011; Sun et al., 2014), while individual overexpression of *CHS* and *ANS* promoted anthocyanin production in *Arabidopsis* and red sage (*Salvia miltiorrhiza*), respectively (Zhang et al., 2018; Li et al., 2019). The combined overexpression of key anthocyanin structural genes affects anthocyanin accumulation in flower species, e.g. rose (Katsumoto et al., 2007) and orchids (Zhou et al., 2021).

Dietary anthocyanins are well regarded for their health benefits, such as providing neuroprotection, lowering cardiovascular disease risks, and having anti-carcinogenic and anti-diabetic properties (Khoo et al., 2017). Blueberry (*Vaccinium* spp.) are known to be one of the best sources of dietary bioactive compounds, with their especially high anthocyanin content purported to confer a wide range of health-promoting benefits (Skrovankova et al., 2015; Ma et al., 2018; Kalt et al., 2020) through their antioxidant and anti-inflammation properties (Riso et al., 2013; Shi et al., 2017; Ma et al., 2018). It has been reported that the type of glycoside moiety conjugated to the anthocyanidin aglycone affects anthocyanin bioavailability (Zhao et al., 2014). However, findings on the effect of each type of glycosylation are inconsistent and this suggests that the sugar/anthocyanidin combination might play a determining role (McGhie et al., 2003; Ichiyanagi et al., 2006; Milbury et al., 2010). Bilberry (*V. myrtillus*) accumulates up to a 4-fold higher concentration of anthocyanins compared with blueberry (Kalt et al., 1999), and is therefore highly valued by the pharmaceutical and dietary supplement industries for their superior health benefits and potential applications due to their high content of bioactive compounds (Camire, 2002; Chu et al., 2011).

Bilberry and the blueberries Northern Highbush (*V. corymbosum*) and Rabbiteye (*V. virgatum*) contain 15 types of anthocyanins distributed over five anthocyanidin groups (cyanidin, peonidin, delphinidin, malvidin and petunidin), with malvidins and delphinidins being the most abundant in blueberry, and cyanidins and delphinidins in bilberry (Kader et al., 1996; Kalt et al., 1999; Dare et al., 2022). While blueberry and bilberry anthocyanidins are commonly linked with glucose, galactose and arabinose, the ratios of the respective glycosides are reported to be species-specific for blueberries (Fang, 2015; Günther et al., 2020; Mengist et al., 2020) and ecotype-specific for bilberry (Dare et al., 2022). Across geographically distinct bilberry populations, the glycosylation pattern varies depending on the region of origin. Such distinct variation in glycosylation pattern has been suggested as a feature for geographical authentication (Primetta et al., 2013).

Despite being one of the richest sources of anthocyanins in common fruits (Kalt et al., 2020), cultivated blueberry species such as Northern Highbush and Rabbiteye blueberry produce anthocyanins only in the fruit skin but not the fruit flesh. In contrast, the undomesticated European bilberry species (*V. myrtillus* L.) has a deep blue colour and pigmented flesh (Riihinen et al., 2008).

Based on the integration of metabolomics and transcriptomics data in blueberry skin and flesh during fruit development, Günther et al. (2020) showed the co-correlation between transcript abundance of blueberry genes *VcCHS1*, *VcANS* and *VcUFGT2* with the accumulation of anthocyanin during blueberry fruit development. In particular, there was a significant decrease in *CHS* and *ANS* expression (between 5- and 15-fold lower) in non-anthocyanic fruit flesh of ripe blueberry fruit when compared with pigmented fruit skin (Günther et al., 2020). A study on wild albino bilberry with highly reduced anthocyanin accumulation found that gene expression of *CHS*, *ANS* and *UFGT* was 7.6, 11 and 33-fold lower, respectively, than in non-albino berry (Zorenc et al., 2017). Similarly, a study on a pink bilberry mutant with lowered anthocyanin accumulation also found a 100-fold decrease in transcript levels of *CHS*, *ANS* and *UFGT* compared with wild-type (Die et al., 2020). Such findings suggest that these three genes may act as bottlenecks in the pathway. These genes, however, have not been functionally characterised in *Vaccinium* before. We hypothesise that there is more than one rate-limiting step in the flavonoid pathway, affecting anthocyanin concentration. A combined effect from a set of these structural genes may be the key to determine the accumulation of anthocyanins. To investigate their roles driving anthocyanin composition and concentration, *CHS*, *ANS* and *UFGT* were cloned from bilberry and blueberry. We then employed the rapid *in vivo* transient expression system using different combinations of these genes in two different model systems (*N. benthamiana* and strawberry) to assess changes in anthocyanin production.

## Methods

### Gene expression analysis

#### Fruit material

Blueberry fruit were harvested from cultivated collections of tetraploid *V. corymbosum* ‘Nui’ (Plant & Food Research, Motueka, New Zealand) (Günther et al., 2020). Three different developmental stages, S5 to S7, were sampled (Zifkin et al., 2012). Bilberry fruit were harvested from wild *V. myrtillus* L. plants in Kvaløya, Norway (Dare et al., 2022). Fruit from three different developmental stages, S3 to S5 (Karppinen et al., 2016), were included in the sampling process. Collected berries were snap-frozen using liquid nitrogen. Frozen berry skins were separated as much as possible from the flesh using a scalpel on dry ice to allow skin-enriched samples. Special care was taken to avoid contaminating fruit flesh samples with anthocyanins from fruit skin. Separated tissues were homogenized in liquid nitrogen to a fine powder using a grinder (IKA A11 basic mill) and kept at −80°C.

#### RNA extraction and cDNA synthesis

RNA was extracted from pulverised fruit tissues (150 mg) using the Spectrum™ Plant Total RNA extraction Kit (Sigma Alrich). RNA concentration was quantified using the DeNovix DS-11 FX Spectrophotometer/Fluorometers (Denovix^®^). Gel electrophoresis was performed to confirm the integrity of the extracted RNA. cDNA synthesis was carried out on 1 μg of extracted RNA using the Quantitect^®^ Reverse Transcription Kit (QIAGEN) with inbuilt gDNA removal step. Control samples without added RT enzyme (-RT) were included for each tissue type to check for possible gDNA contamination.

#### Quantitative real-time PCR

Quantitative real-time reverse transcription PCR (qRT-PCR) was performed on cDNA (1:20 dilution) from blueberry and bilberry fruit skin and flesh tissues. Reactions were performed in four technical replicates to a total volume of 10 μL (5 μL LightCycler SYBR Green I Master, 0.5 μL of 10 μM forward and reverse primer mix, 2.5 μL diluted cDNA (1:20) and 2 μL nuclease free water). The LightCycler^®^480 system and software (version 1.5, Roche) was used for amplification at the following conditions: 95°C for 5 min, followed by 50 cycles consisting of 95°C for 10 s, 60°C for 10 s and 72°C for 20 s, then continuous fluorescence data acquisition from 65 to 95°C for melting curve detection. The expression of six anthocyanin biosynthetic genes *CHALCONE SYNTHASE* (*CHS1* and *CHS2*), *DIHYDROFAVONOL REDUCTASE* (*DFR*), *FLAVANONE 3-HYDROXYLASE 1* (*F3H1*), *ANTHOCYANIDIN SYNTHASE* (*ANS*) and *UDP-GLYCOSE: FLAVONOID-3-O-GLYCOSYLTRANSFERASE 2* (*UFGT2*) as well as the anthocyanin-related TF *MYBA1* was analysed. For both blueberry and bilberry, *GLYCERALDEHYDE-3-PHOSPHATE DEHYDROGENASE* (*GAPDH*) and *SAND FAMILY PROTEIN* (*SAND*) were used as reference/house-keeping genes. Primer sequences (**Supplementary Table 1**) were designed based on RNA-Seq data from Günther et al. (2020) and primer efficiency was determined using the LinReqPCR software (Tuomi et al., 2010). A melting curve analysis was performed using the LightCycler^®^480 software to verify the amplification of only a single reaction product. Relative gene expression compared to housekeeping genes was calculated based on method described by André et al. (2009).

### Gene identification and phylogenetic analysis

The open reading frame (ORF) sequences for *VcCHS1* (maker-VaccDscaff35-augustus-gene-1.21-mRNA-1), *VcANS* (maker-VaccDscaff46-augustus-gene-11.23-mRNA-1) and *VcUFGT2* (maker-VaccDscaff6-augustus-gene-420.36) originated from the tetraploid *V. corymbosum* genome (Colle et al., 2019) as described in (Günther et al., 2020). Bilberry homologues were identified from the *V. myrtillus* reference genome (Wu et al., 2021) using the Basic Local Alignment Search Tool (BLAST) algorithm.

Protein sequences encoded by these candidate genes from blueberry, were used to search for orthologues from publicly available proteome data from *Arabidopsis* (Berardini et al., 2015) and five other red fruits – *Fragaria vesca* (strawberry) V4.0 a1 genome database (Edger et al., 2018), *Actinidia chinensis* (kiwifruit) Red5 genome database (Pilkington et al., 2018), *V. macrocarpon* (cranberry) (Polashock et al., 2014), *Vitis vinifera* (grape) (Enrico, 2007), *Rubus occidentalis* (raspberry) (VanBuren et al., 2018), and *Solanum lycopersicum* (tomato) (Tomato Genome Consortium, 2012). Additional protein sequences were retrieved from other species using protein BLAST from non-redundant protein sequences (nr) database (https://blast.ncbi.nlm.nih.gov/Blast.cgi). Multiple nucleotide and amino acid sequence alignments and phylogeny trees were created using Geneious software version 10.2.5 (Kearse et al., 2012). The CHS phylogenetic tree was constructed with 54 amino acid sequences from 39 plant species. The ANS phylogeny consisted of 51 sequences from 41 species. LDOX sequences from grape, tobacco, apple, kiwifruit and blueberry were also included in this analysis because of their high similarity in sequence and function of LDOX and ANS (Jun et al., 2018; Lafferty et al., 2022a). There were 62 sequences from 52 species included in the UFGT phylogenetic tree. Consensus phylogenetic trees were constructed using the Neighbor-Joining method with bootstrap at 10,000 replicates. Phylogenetic tree visualisation was generated using iTOL v6 (Letunic and Bork, 2021).

### Gene cloning

For *VmCHS1* (GenBank accession OP966665) and *VmANS* (GenBank accession OP966664), total RNA was extracted from bilberry leaf and used for cDNA synthesis (RNA extraction and cDNA synthesis were as described above). Gene-specific primers were designed to match the sequence of both bilberry and blueberry (**Supplementary Table 2**). High-Fidelity DNA Polymerase (iProof, BioRad) was used for ORF amplification, introducing *Hind*III and *Bam*HI restriction sites at the 3’ and 5’ end, respectively, at optimised PCR conditions (98°C for 30 s, 30 cycles at 98°C for 10 s, 61°C for 15 s and 72°C for 1.5 min, followed by a final extension at 72°C for 10 min). The Zymoclean Gel DNA Recovery Kit (Zymo Research) was used for purifying *VmCHS1* and *VmANS* fragments, respectively, which were then cloned into the pJET1.2/blunt cloning (pJet) vector and transformed into *Escherichia coli* DH5α competent cells through heatshock. *E. coli* cells were grown on Luria-Bertani (LB) media containing ampicillin (50 μg/mL) to amplify the plasmid for Sanger-sequencing (Macrogen). Sequence identity was confirmed against the reference sequence of the *V. myrtillus* reference genome (Wu et al., 2021). The ORFs of *VmCHS1* and *VmANS* were subsequently cut using restriction enzymes (*Bam*HI and *Hind*III, NEB) and cloned into the vector pGreen0029-62-SK (pGreen) (Hellens et al., 2000) using T4 DNA ligase (Thermo Scientific). After amplification in *E. coli* DH5α cells on LB-media with kanamycin (50 μg/mL), rifampicin (50 μg/mL) and gentamycin (10 μg/mL), the Zyppy™ Plasmid Miniprep Kit (Zymo Research) was used for plasmid purification and the correct orientation of the gene confirmed by Sanger sequencing. Recombinant pGreen vectors were transformed into *Agrobacterium tumefaciens* (*Agrobacterium*) strain GV3101 (carrying the pSoup helper vector) using electroporation (2.5 kV, capacitance 25 μFd, resistance: 400 Ohms with pulse time of 7-9 ms). *Agrobacterium* were recovered in Super optimal broth with catabolite repression (SOC) media after transformation and used for subsequent transient expression assays.

For *VcUFGT2*, double-stranded gblocks™ DNA fragments (Integrated DNA Technologies) were synthesised for the entire *VcUFGT2* (GenBank accession OP966666, maker-VaccDscaff6-augustus-gene-420.36, Günther et al., 2020) ORF (Integrated DNA Technologies) and used as template (1.5 ng/µl) for amplification using High-Fidelity DNA Polymerase (iProof, BioRad) with gene-specific primers, introducing attB-attachment sites for subsequent Gateway^®^ cloning. As Forward Gateway primer 3’ *VcUFGT2* and as Reverse Gateway primer, 5’ *VcUFGT2* was used (**Supplementary Table 2**). PCR conditions were: 98°C for 30 s, 10 cycles at 98°C for 10 s, 71°C for 15 s and 72°C for 30 sec, followed by a final extension at 72°C for 5 min. The gel purified (Zymoclean Gel DNA Recovery Kit, Zymo Research) PCR product (50-100 ng) was then cloned overnight (20°C) into pDONR221™ vector (150 ng) using BP Clonase™ II enzyme mix in a 10 µL reaction volume. The pHex destination vector (150 ng) and 2µL LR Clonase™ II enzyme mix was then added to and aliquot of the reaction mixture to a final volume of 10 µL and incubated overnight (20°C). OneShot™ Top10 chemically competent *E. coli* cells (Invitrogen™) were transformed with 2 μL of the reaction and plated onto selective LB-agar containing spectinomycin (25 μg/mL). Sanger-sequencing (Macrogen) was used to verify the *VcUFGT2* sequence from eight random colonies after amplification of the plasmid and subsequent purification.

### *Agrobacterium*-mediated transient transformation in tobacco and strawberry

#### Plant materials

Tobacco plants (*N. benthamiana*, ecotype *‘*Northern Territory’, kindly provided by Prof. Peter Waterhouse at Queensland University of Technology) were grown from seed. Mature strawberry plants (*Fragaria × ananassa* ‘Camarosa’) were purchased from a garden centre in Auckland, New Zealand. These plant materials were grown in a containment glasshouse at Plant and Food Research (Auckland, New Zealand) at standard glasshouse conditions (20-24°C, 12 hr day lighting condition) for transient gene expression assays. *N. benthamiana* plants were used at 5.5 weeks old and the two youngest leaves chosen for at least six infiltration patches. Mature strawberry fruit (with fully developed seeds) were infiltrated at the initial reddening stage as described by (Jia et al., 2020) (**Supplementary Figure 1A**).

#### *Agrobacterium*-mediated transient transformation

*VcMYBA1* (GenBank accession MH105054.1; Plunkett et al., 2018), *MdCHS2* (GenBank accession EB120544.1; Dare et al., 2013)*, AmF3´5´H* (GenBank accession OP963713; Peng et al., 2019) and the reporter gene *GUS* (Peng et al., 2020) as control have been previously reported. *Agrobacterium w*ere grown on LB-agar containing rifampicin (50 μg/mL), gentamycin (10 μg/mL) and kanamycin (50 μg/mL, for *VmCHS1* and *VmANS*) or spectinomycin (25 μg/mL, for *VcMYBA1, VcUFGT2, MdCHS2, AmF3´5´H* and *GUS*) at 28°C overnight to achieve exponential growth phase. Using a 10 μL inoculation loop, *Agrobacterium* cells were then suspended in infiltration buffer (10 mM magnesium chloride, 10 μM acetosyringone) to appropriate OD_600_ levels and incubated at 28°C for 2 h before infiltration. Different combinations of *MdCHS2, VmCHS1*, *VmANS* and *VcUFGT2* (OD_600_ = 0.3 each) were mixed in excess with minimal amounts of the TF *VcMYBA1* (OD_600_ = 0.03) to activate the expression of the anthocyanin biosynthesis pathway in tobacco leaves. *GUS* was used as a negative control in both tobacco and strawberry, and to ensure equal final OD_600_ = 1.2 and 35S promoter activity of all mixtures. Each *N. benthamiana* plant was considered a separate biological replicate and infiltrated individually to avoid contamination between treatments. At least three biological replicates were harvested per treatment. Gene expression of some infiltrated genes in *N. benthamiana* leaves was confirmed using end-point PCR (**Supplementary Table 3**; **Supplementary Figure 2**).

AmF3´5´H was functionally tested using transient overexpression in *N. tabacum* leaves as described by Peng et al. (2019). Co-infiltration of *VcUFGT2* with both *VcMYBA* (Plunkett et al., 2018) and *AmF3´5´H* was used in strawberry to produce delphinidin-based anthocyanin, which is not naturally occurring in strawberry. Strawberry fruit were infiltrated by injections of approximately 1 mL mixed culture into the fruit flesh. Infiltrated fruits were harvested when fully ripened after five to seven days (**Supplementary Figure 1B**). Only visibly transformed fruit tissues (showing dark colour development) were sampled. Each treatment was harvested in at least four biological replicates with each replicate comprising a pool of three to four berries. All infiltrated tissues were snap-frozen in liquid nitrogen and stored at -80°C.

### Anthocyanin extraction and quantification from plant tissues

#### High-Performance-Liquid chromatography

Freeze-dried plant tissue (5 to 15 mg) was extracted in methanol containing 0.1% (v/v) HCl (500 μL) for 3 h in the dark at room temperature with hourly vortexing and then centrifuged at 15,000 rpm for 10 min. The supernatant was transferred, evaporated to dryness using a CentriVap^®^ concentrator (Labconco, CA, USA) and resuspended in 20% (v/v) methanol (400 µL). The solutions were vortexed and centrifuged for 10-30 seconds at 15,000 rpm. Supernatant was filtered into a new Eppendorf tube using 1 mL syringe and RC membrane, pore size 0.45 μm, 15 mm syringe filter (Phenomenex^®^) prior to analysis.

The HPLC system used was a Dionex UltiMate 3000 UHPLC coupled to a Photodiode Array (PDA) detector (Dionex, Sunnyvale, CA, USA). Anthocyanins were separated on an Acclaim PolarAdvantage II (PA2), 4.6 × 150 mm, 5 µm, analytical LC column (Thermo Fisher Scientific), maintained at 35°C. The solvents were (A) 95:5 water:formic acid v/v and (B) acetonitrile (flow rate, 0.35 mL/min). The initial mobile phase, 100% A, was ramped up linearly to 93% A at 5 min, then to 88% A at 10 min, and 65% A at 25 min, followed by a column flush at 0% A before resetting to the original conditions. The sample injection volume was 5 µL and PDA detection was from 200 to 600 nm. Anthocyanin concentrations were quantified as cyanindin-3-*O*-glucoside equivalents using standard curves constructed from serial dilutions of cyanindin-3-*O*-glucoside (concentration range from 15 to 150 μg/μL) with PDA detection at 520 nm. Total anthocyanin concentration was normalised to μg total anthocyanin/g dry weight (DW).

#### Liquid chromatography-mass spectrometry (LC-MS)

Freeze-dried plant tissue (15 mg) was extracted in methanol+1% formic acid (1 mL) for 3 h at room temperature and vortexed every hour. Cyanidin rutinoside (4 µg/mL^)^ was added as internal standard. The supernatant was transferred after centrifugation (15 min; 15,000 rpm), dried under nitrogen and re-suspended in 5% acetonitrile (100 µL) for analysis.

The samples were analysed by LC-MS using an LTQ linear ion trap mass spectrometer fitted with an ESI interface (electrospray voltage: 5 kV, source temperature: 275°C) (ThermoFisher Scientific, San Jose, CA, USA) coupled to a Dionex Ultimate 3000 UHPLC and PDA detector (Dionex, Sunnyvale, CA, USA). Anthocyanins were separated on an Accucore 2.6 µm C18 150 × 2.1 mm column (Thermo Scientific, CA, USA). The flow rate was 0.4 mL/minute, column temperature 40°C and injection volume was 2 µL. The solvents were (A) 90:10 water:formic acid v/v and (B) 22.5:22.5:45 methanol:acetonitrile:water + 10% formic acid v/v/v. The initial mobile phase, 97% A was held for 0.5 min, then ramped up linearly to 70% A at 22.5 min, and 50% A at 25.5 min, followed by a column flush at 30% A before resetting to the original conditions. The MS data were acquired in the positive mode. The Xcalibur software was used for peak integration and concentrations were calculated in equivalence to cyanidin-3-*O*-glycoside, based on a standard curve of serial dilution.

### Statistical analysis

All statistical analyses were conducted using R 4.0.3 version “Bunny-Wunnies Freak Out” (R Core Team, 2020). The Shapiro-Wilk test and histograms plots were used to inspect distribution of data populations using the ‘MVN’ package (Korkmaz et al., 2014). Where normality was rejected, Box-Cox transformation of the data was performed. Standard parametric analysis was performed, namely Student’s t-test (Student, 1908) for qRT-PCR data and for transient expression data one-way analysis of variance (ANOVA) followed by Tukey-Kramer Honest significant difference (Yandell, 2017) *post-hoc* test was used. All analyses were conducted at α = 0.05 using the Benjamini-Hochberg correction (Benjamini and Yekutieli, 2001) for multiple comparisons.

## Results

### RT-qPCR analysis shows that *ANS*, *CHS1* and *UFGT2* expression correspond with anthocyanin production in both blueberry and bilberry

**Figure 1:**
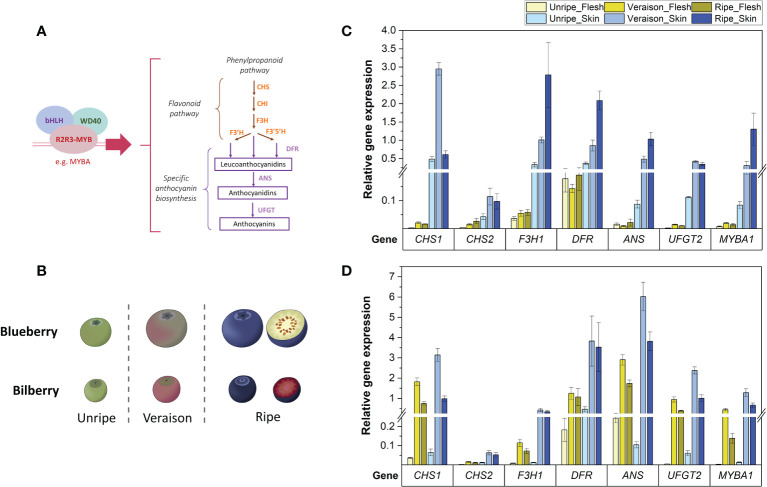
**(A)** The anthocyanin biosynthesis pathway showing structural genes and the MBW complex controlling the pathway. **(B)** Developmental stages used for RT-qPCR analysis of blueberry and bilberry fruit. Fruit stages unripe-ripe correspond to blueberry stages S5-7 (Zifkin et al., 2012) and bilberry stages S3-5 (Karppinen et al., 2016). **(C, D)** RT-qPCR analysis of the TF *MYBA1* and structural anthocyanin genes in the flesh and skin of blueberry **(C)** and bilberry **(D)** at three fruit developmental stages (unripe, veraison and ripe). Mean relative expression from at least three technical replicates is shown. Error bars represent ± standard deviation.

Structural genes were previously identified as relevant for anthocyanin biosynthesis in blueberry and bilberry using RNA-Seq (Günther et al., 2020; Lafferty et al., 2022b). To validate their expression before cloning, RT-qPCR was performed on fruit skin and flesh tissues from both blueberry and bilberry at three distinct stages during fruit ripening. Transcript abundances of the three candidate genes *CHS1*, *ANS* and *UFGT2* (**Figure 1A**) was analysed in comparison with the main anthocyanin activator *MYBA1* and additional non-candidate genes from the flavonoid pathway (*CHS2*, *F3H1* and *DFR*). In both *Vaccinium* species, identical primers were used and candidate genes were successfully quantified from pigmented as well as non-pigmented tissues of unripe, veraison (start of skin colour change) and ripe fruit (**Figure 1B**).

In blueberry, the relative expression of all tested genes was significantly higher in fruit skin compared with fruit flesh across all fruit developmental stages (p-value < 2.5 x 10^-7^) and was highest in the skin of pigmented (veraison and ripe) fruit (**Figure 1C**). Transcript abundances of *MYBA1*, *F3H1*, *DFR* and *ANS* were highest in ripe fruit skin (p-value < 0.009). The expression level of *UFGT2* remained at similar levels between both veraison and ripe stages (p-value = 0.53). Meanwhile, transcript abundance of *CHS1* was approximately six-times higher in fruit skin at the veraison stage compared with both unripe and ripe fruit (p-value < 6.4 x 10^-4^). In bilberry, gene expression of tested flavonoid genes was also highest in fruit skin of ripening (veraison and ripe) fruit (**Figure 1D**) and reflecting *MYBA1* expression levels.

The expression level of *CHS1*, *ANS*, *UFGT2* and *MYBA1* were considerably higher in blueberry fruit skin (between 30- to 103-fold) than in flesh tissue (**Figure 1C**). In contrast to blueberry, bilberry produced anthocyanins in fruit flesh and transcript abundance of the three candidate genes and *MYBA1* was on average only 2.3-fold higher in fruit skin when compared with flesh (**Figure 1D**). Such changes in the fruit skin to flesh expression ratio in the two *Vaccinium* species confirmed the association between candidate gene expression and anthocyanin production.

In contrast, the additional *CHS* isoform (*CHS2*) and *DFR* only showed an approximately 3.4-fold increase in expression in fruit skin compared with fruit flesh for both blueberry and bilberry (**Figure 1C, D**). *DFR* also showed high abundance in the non-anthocyanic blueberry fruit flesh, which was on average 44-fold higher at the unripe fruit, and 10-fold higher at the veraison and ripe fruit stage than the other tested genes. *CHS2*, conversely, showed relatively low expression in the fruit skin and flesh of both bilberry and blueberry (**Figure 1C, D**). *F3H1* had a high fruit skin/flesh ratio similar to the three candidate genes and *MYBA1* (27-fold) in blueberry (**Figure 1C**). However, in bilberry, the expression of *F3H1* was on average 8-fold lower than *CHS1*, *ANS* and *UFGT2* in both the pigmented fruit flesh and skin (veraison and ripe stages) (**Figure 1D**).

In summary, *CHS1* was the most highly expressed isoform for anthocyanin production in the fruit of both *Vaccinium* species. *ANS* and *UFGT2* had the most similar expression pattern to the TF *MYBA1* regarding the difference between fruit skin/flesh in blueberry and between blueberry/bilberry fruit flesh. All candidate genes were highly expressed in the fruit skin of berries of both species.

Based on the gene expression analyses, *CHS*, *ANS* and *UFGT2* were confirmed as suitable gene candidates modulating anthocyanin production in both blueberry and bilberry. Bioinformatic analysis was performed for each of these three genes to further study their sequence similarities across different plant species in detail. First, nucleotide sequences of *CHS*, *ANS* and *UFGT2* from both bilberry and blueberry were identified to investigate the similarity between the two species. Two putative *VmCHS* versions, one *VmANS* and four *VmUFGT*s were retrieved from the bilberry genome (Wu et al., 2021) using the nucleotide sequences from blueberry (*VcCHS1, VcANS* and *VcUFGT2)* as previously identified by Günther et al. (2020). For *CHS* and *UFGT* from bilberry, the sequences with the highest similarity with *VcCHS1* and *VcUFGT2* (*VmCHS1* and *VmUFGT2*) were chosen for further experimental characterisation. The previous annotation as *anthocyanidin-3-O-glycosyl transferase 2* (*A3GT2*) has not been experimentally validated, therefore, *UFGT* was used as a general term. For all three genes, the bilberry and blueberry sequences were near identical with identities ranging from 95% to 99% for both nucleotide and deduced amino acid sequences.

**Figure 2:**
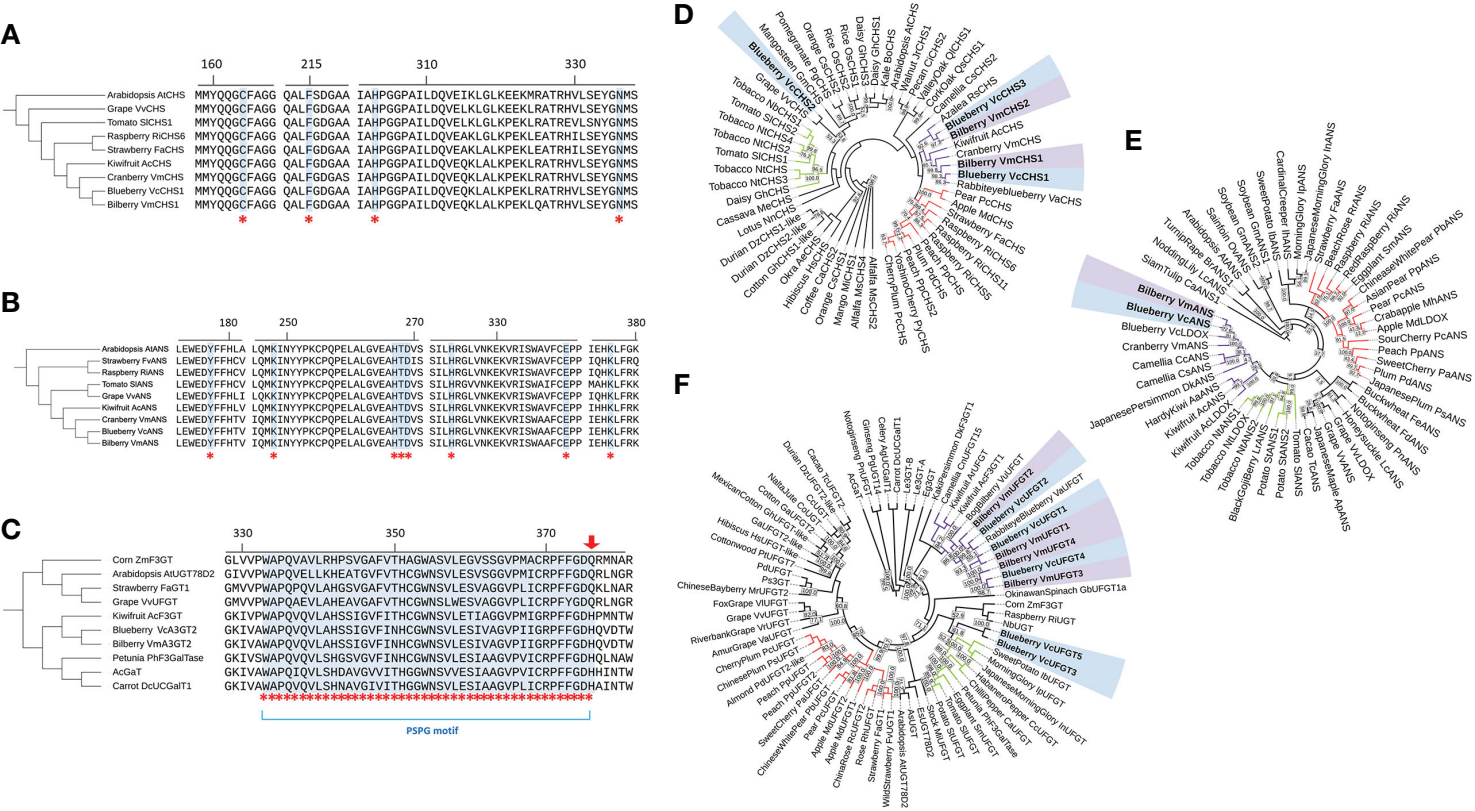
Active site alignment and phylogenetic tree of the protein sequence of CHS **(A, D)**, ANS **(B, E)** & UFGT2 **(C, F)**. **(A-C)** Active site residues are highlighted and indicated by asterisks and are referred to in the text. Arrow indicates conserved residue for glucosyltransferases/galactosyltransferases. Residue numbering is based on blueberry sequence. **(B-F)**: Blueberry and bilberry sequences are in bold and highlighted (blueberry – blue; bilberry – purple). Clusters of the three most represented taxonomic orders are indicated by branch colour (Rosales – red; Solanales – green; Ericales – purple; others - black). Phylogenetic trees were constructed using the Neighbour-Joining algorithm with 0.5% topology support threshold. Bootstrap analysis was performed at 10,000 replicates, values > 50% are shown on the tree. Sequence accession numbers are indicated in **Supplementary Tables 2**, **3** and **4**.

To evaluate the relevance of CHS1, ANS and UFGT2 in the anthocyanin biosynthesis pathway, the amino acid sequences from both blueberry and bilberry were aligned with homologous sequences from other plant species, including *Arabidopsis* and anthocyanin-producing fruits. For UFGT, seven anthocyanin-producing species with functionally characterised flavonoid-3-glycosyltransferes activity were included (Furtek et al., 1988; Miller et al., 1999; Lunkenbein et al., 2006; Offen et al., 2006; Kubo et al., 2007; Montefiori et al., 2011). Alignment results of CHS amino acid sequences showed that four CHS active site residues, Cys164, Phe215, His303 and Asn336 (Jez et al., 2001), were fully conserved and identical in all sequences considered (**Figure 2A**). For ANS, all eight active site residues identified in AtANS (Wilmouth et al., 2002) were fully conserved in blueberry, bilberry and seven other species (**Figure 2B**). Similarly, the PSPG (plant secondary product glycosyltransferases) motif (Hughes and Hughes, 1994) of UFGT was fully conserved between the considered sequences (**Figure 2C**), except for the last residue of the motif. VcUFGT2 and VmUFGT2 have a histidine residue in this position which was previously found to be highly conserved among galactosyltransferases, such as the kiwifruit AcF3GT (Montefiori et al., 2011). This is in contrast with glucosyltransferases such as the grape VvUFGT (Offen et al., 2006), which has a conserved glutamine residue at the end of the PSPG motif.

The phylogenetic relationship between bilberry and blueberry amino acid sequences with other plant species was investigated for each candidate gene. CHS and ANS were highly conserved across the different plant species considered. UFGT was less conserved, with the majority of sequences showing 40-50% identity to each other. For all CHS, ANS and UFGT, most sequences from the same taxonomic order clustered well with each other (**Figure 2D-F**). Most sequences from the order Ericales clustered together (purple clusters) with the exception of VcUFGT3, VcUFGT5 and VcCHS2. Within the Ericales subcluster, *Vaccinium* species showed the highest homology. Especially, blueberry sequences (highlighted blue) were most closely related to their corresponding homolog in bilberry (highlighted purple) (e.g. VcCHS1 and VmCHS1, VcUFGT2 and VmUFGT2) (**Figure 2D, F**). Overall, the high similarity and close relationship between the blueberry and bilberry candidate genes validated the use of cloned genes from either species for testing their function in *Vaccinium* species using transient expression.

### Overexpression of VmANS increases anthocyanin concentration

*Agrobacterium-*mediated infiltration assays in *N. benthamiana* (Hellens et al., 2005) were used to characterise the function of *VmCHS1*, *VmANS* and *VcUFGT2* in modulating anthocyanin biosynthesis *in vivo*. Co-infiltration with *VcMYBA* at an OD_600_ ratio of 1:10 to other genes was found to be suitable for further assaying activities by providing a low but visible and quantifiable background of anthocyanin production in leaves.

**Figure 3:**
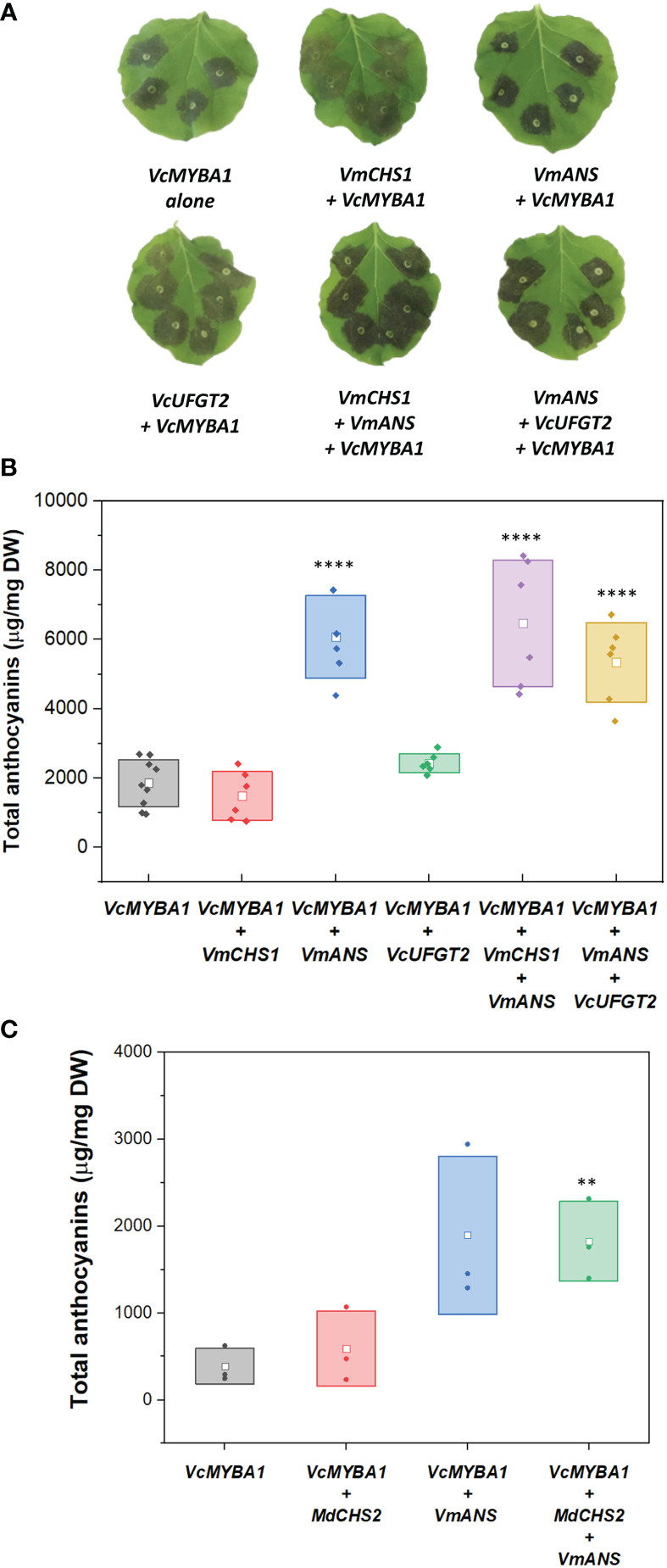
**(A)** Images of *N. benthamiana* leaves infiltrated with different combinations of *VcMYBA1, VmCHS1*, *VmANS*, and *VcUFGT2*. The combinations of genes used are indicated below each leaf image. Images were taken at 5 days post-infiltration. **(B)** Total anthocyanins from leaves infiltrated with different gene combinations. **(C)** Total anthocyanins from leaves infiltrated with *MdCHS2*. Filled dots represent data points from each biological replicate. Empty squares represent the mean concentration value of each treatment. Boxes represent the mean ± one SD from at least three biological replicates. Statistical analysis from pairwise comparison with *VcMYBA1* control samples (Student’s t-test, N ≥ 3, α = 0.05), p-value < 0.005 is indicated by **; p-value < 10^-4^ is indicated by ****.

First, effects on anthocyanin production by single candidate gene overexpression assays were assessed. *VcMYBA1*-only samples produced anthocyanin concentration of 1.81 ± 0.88 mg/g DW (**Figure 3**). Compared to the infiltration of *VcMYBA1* alone, co-infiltration with *VmCHS1* did not result in a significant change in anthocyanin production (p-value = 0.985). This was observed both visually, in similar pigmentation intensity of the infiltrated sites (**Figure 3A**), and in the quantification of anthocyanin concentration using HPLC with PDA detector (**Figure 3B**). An additional experiment was conducted using co-infiltration of *VcMYBA1* and the apple *MdCHS2*, which has been previously functionally characterised (Dare et al., 2013). However, transient expression of *MdCHS2* did not significantly change anthocyanin production either compared with the *VcMYBA1* control (p-values = 0.969, **Figure 3C**). This indicated that neither CHS led to increased anthocyanin production compared with the *VcMYBA1* control in *N. benthamiana*. Similarly, *VcUFGT2* + *VcMYBA1* infiltration resulted in a 1.3 times higher mean anthocyanin concentration (2.43 ± 0.28 mg/g DW, **Figure 3B**) compared to with the *VcMYBA1* control. However, this was not a significant increase (p-value = 0.906). In contrast, infiltration of *VmANS* + *VcMYBA1* resulted in a significant 3.57-fold increase in total anthocyanin compared with the *VcMYBA1* control samples with 6.08 ± 1.20 of mg total anthocyanin/g DW produced (p-value < 10^-4^).

Co-infiltrations of different candidate genes with *VmANS* were tested to evaluate possible combination effects on anthocyanin production. When *VmANS* was co-infiltrated with either *VmCHS1* or *VmUFGT2*, there was a significant increase (p-value < 10^-4^) in anthocyanin production when compared with the *VcMYBA* control (**Figure 3B**). In particular, combinations with *VmANS (*i.e. *VcMYBA1* + *VmCHS1* + *VmANS* and *VcMYBA1* + *VmANS* + *VcUFGT2*) resulted in anthocyanin concentrations of 5.34 ± 1.13 mg/g DW and 6.47 ± 1.20 mg/g DW, respectively. However, there was no significant difference between the two combinations (p-value = 0.46). Therefore, the same trend as single candidate gene infiltrations regarding changes in anthocyanin concentration was observed when different gene combinations were tested. This suggested that *VmANS* was the only candidate gene with a strong effect in increasing anthocyanin concentration in this assay. These results reflected the visual evaluation of infiltration sites, which were darker in colour compared to that of the *VcMYBA* control and *VmCHS1/VcUFGT2* infiltrations (**Figure 3A**). Co-infiltration of *VmCHS1*, *VmANS* and *VcUFGT2* with other candidate genes without *VcMYBA* was also performed, but no anthocyanin was produced (**Supplementary Figure 3**).

**Figure 4:**
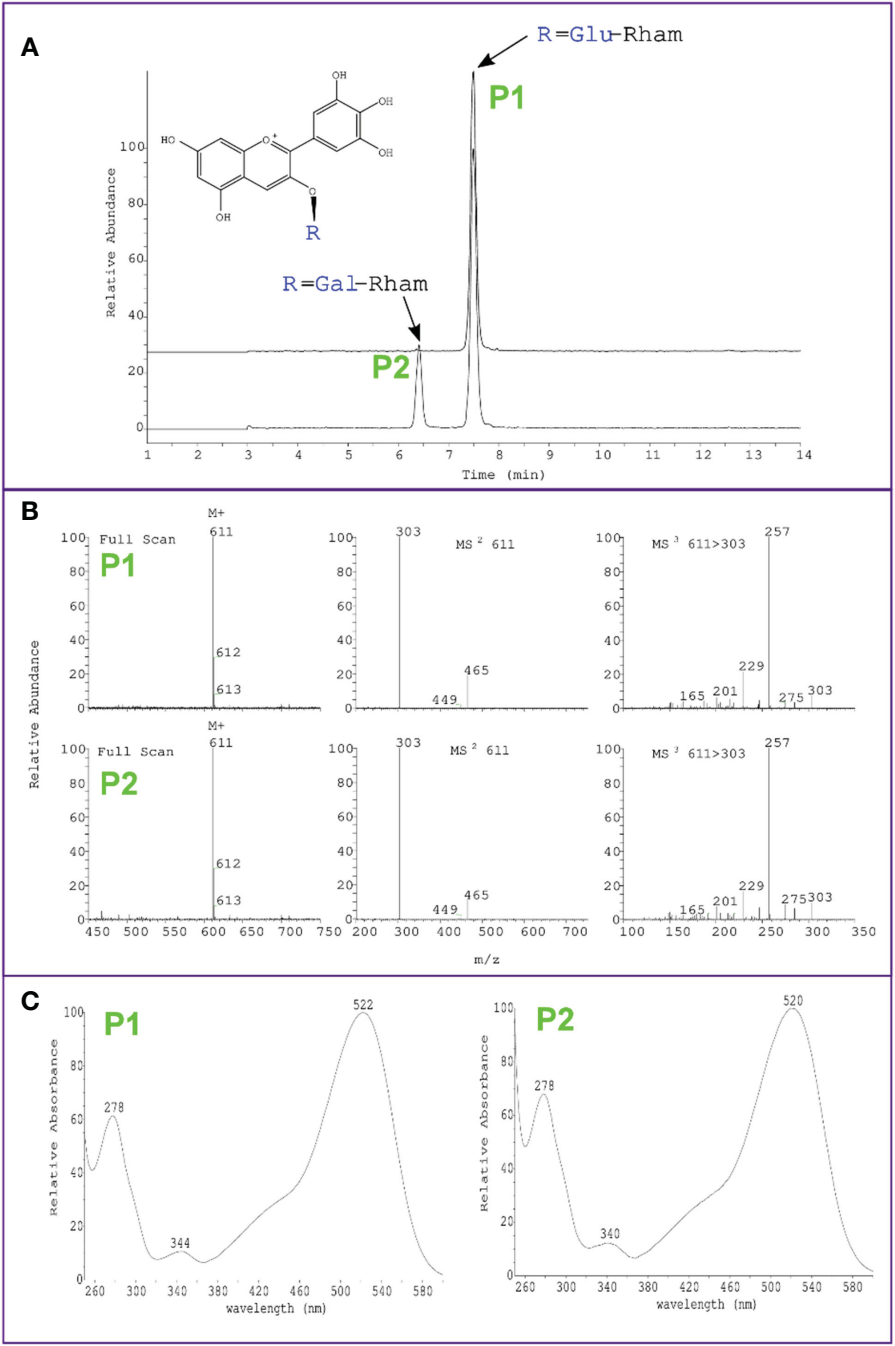
Identification of delphinidin-3-*O*-galactorhamnoside production in *N. benthamiana* leaves using *Agrobacterium*-mediated transient overexpression of *VcMYBA1* + *VcUFGT2*. **(A)** Chromatogram showing peak detection at 530 nm and earlier retention time of delphinidin-3-*O*-galactorhamnoside (P2) compared with delphinidin-3-*O*-rutinoside (P1) of *VcMYBA1* + VcUFGT2 (bottom trace) in comparison to *VcMYBA1* + *VmANS* as control (top trace). **(B)** Mass spectra of positive ion mode full scan MS, MS^2^ and MS^3^ fragmentation of the respective selected main ion, showing loss of rhamnose and hexose in MS^2^ and fragmentation of the delphinidin aglycone in MS^3^. Molecular weight and fragmentation spectra are identical for P1 and P2, indicating that both anthocyanins have the same chemical formula and are therefore stereoisomers. **(C)** Full UV-Vis spectral scan, showing typical absorption spectrum of delphinidin for both compounds.

Interestingly, while overexpression of *VcMYBA1, VmCHS1* or *VmANS* resulted in the biosynthesis of a single type of anthocyanin (delphinidin-3-*O*-rutinoside/delphinidin-3-*O*-glucorhamnoside), overexpression of *VcMYBA1* with *VcUFGT2* or *VcUFGT2* + *VmANS* resulted in an additional anthocyanin peak, which was identified as delphinidin-3-*O*-galactorhamnoside using LC-MS (**Figure 4**). This supports a primary function for UFGT2 in modulating anthocyanin composition, instead of concentration, in *N. benthamiana* and suggests a high affinity for utilising UDP-galactose as substrate.

### UFGT2 alters the composition of anthocyanin produced

As observed in **Figure 4**, overexpression of *VcUFGT2* in *N. benthamiana* resulted in the production of an additional anthocyanin linked with galactorhamnoside, which is not naturally occurring in either *Nicotiana* or *Vaccinium*. The types of anthocyanins produced are likely dependent on both the affinity of the glycosyltransferase and the availability of the respective anthocyanidin and UDP-sugar substrates (Montefiori et al., 2011). To further investigate the role of *VcUFGT2* in inducing the anthocyanin profile common to blueberry and bilberry in a fruit system, ‘Camarosa’ strawberry was chosen as a fruit transient overexpression system. Ripe ‘Camarosa’ strawberry naturally produces only two types of anthocyanins, pelargonidin-3-*O*-glucoside as the major compound (95%) and cyanidin-3-*O*-glucoside as the minor anthocyanin (5%). Preliminary experiments showed that overexpression of *VcMYBA1* in strawberry fruit significantly increased the amount of cyanidin-3-*O*-glucoside to up to 35% total anthocyanins (**Supplemental Figure 5**).

**Figure 5:**
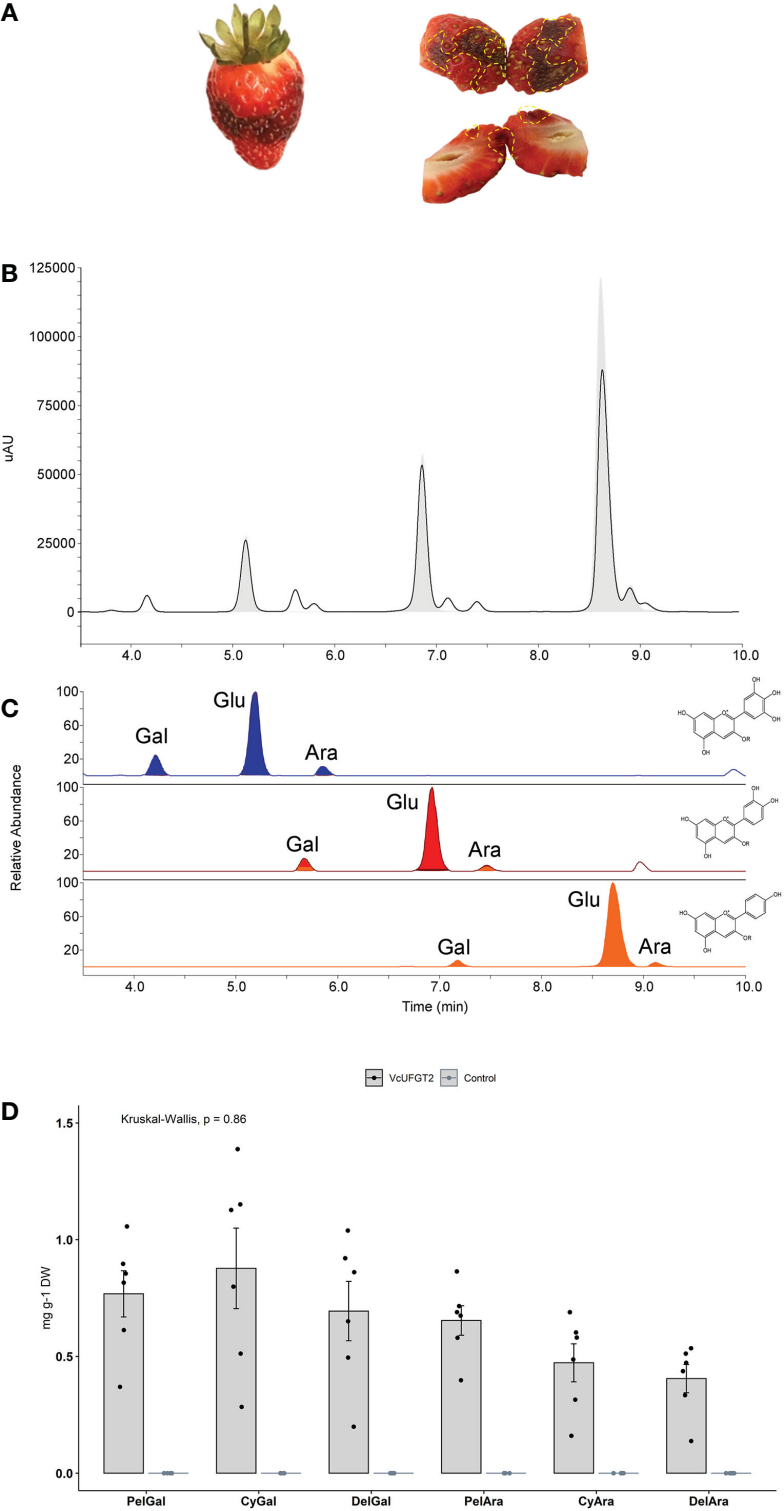
Anthocyanin profile of strawberry fruit overexpressing *VcUFGT2* in a *VcMYBA1* and *AmF3´5´H*–co-expression background. **(A)** Representative images of whole (left) and bisected (right) strawberry fruit infiltrated with *VcMYBA1* + *AmF3´5´H*. Dotted yellow lines highlight transformed tissue showing darken colour that was sampled for further analysis. **(B)** Chromatogram indicating retention times of compounds from control (grey fill) and *VcUFGT2*-infiltrated (not filled) fruit at 530 nm. **(C)** Retention times of MS transitions for delphinidin- (blue; 465, 435), cyanidin- (red; 449, 419) and pelargonidin-derived (orange; 433, 403) anthocyanins. Gal: Galactoside; Glu: Glucoside; Ara: Arabinoside. **(D)** Bargraph visualising mean concentration ± standard deviation (N=6) of anthocyanidin-3-*O*-galactosides and -arabinosides following strawberry transient transformation 5 days after infiltration. While these anthocyanins were not detected in control samples, concentrations of individual anthocyanins were not significantly different in *VcUFGT2*-infiltrated fruit. Pel, Pelargonidin; Cy, Cyanidin; Del, Delphinidin.

In contrast to blueberries and *N. benthamiana*, strawberries do not produce delphinidin-based (blue) pigments due to the absence of FLAVONOID 3´5´ HYDROXYLASE (Song et al., 2015). To functionally test the substrate specificity of VcUFGT2 with respect to the anthocyanidin and UDP-sugar used for biosynthesis in fruit, we aimed to modify the chemical environment in strawberry fruit to include trihydroxylated anthocyanidins. This was achieved by the transient overexpression of *VcMYBA1* with an active kiwifruit (*A. melanandra*) *AmF3´5´H* (Peng et al., 2019) (**Supplementary Figure 5**) resulting in the production of delphinidin-3-O-glucoside, which is not naturally present in strawberry due to the absence of this enzyme. Additionally, modified strawberry produced dark-pigmented patches as a result of delphinidin being produced (**Figure 5A**), thus, providing a visual cue for sampling transformed tissues.

The total amount of anthocyanins and that of the primary anthocyanidin-3-*O*-glucosides did not change significantly in response to *VcUFGT2* overexpression in a *VcMYBA1* and *AmF3´5´H*–co-expression background (**Supplementary Figure 6**). However, six additional anthocyanins were produced, linking each of the respective anthocyanidins to either galactoside or arabinoside (**Figure 5**). Thus, *VcUFGT2* was confirmed in a fruit system to utilise UDP-galactose and UDP-arabinose as substrates to stabilise a range of anthocyanidins as precursors, including pelargonidin, which is not naturally present in blueberry.

## Discussion

In this study, we characterised three genes, *CHS1*, *ANS* and *UFGT2*, from the *Vaccinium* flavonoid pathway leading to anthocyanin biosynthesis, and assessed their role in modulating anthocyanin production both individually and in combination. qRT-PCR was performed for the fruit skin and flesh tissue of blueberry and bilberry, which showed highest expression of *CHS1*, *ANS*, *UFGT2* and the TF *MYBA1* in anthocyanin-rich skin of ripe fruit. The expression of these genes also corresponded to anthocyanin accumulation in both *Vaccinium* species as there was very low expression in blueberry flesh, but substantially higher in bilberry flesh. Additional non-candidate structural genes from the flavonoid pathway (*CHS2*, *F3H1* and *DFR*) included in the analysis, did not exhibit such pattern. Markedly, despite being an isoform of *Vaccinium CHS1*, *CHS2* expression was low in both tissue types throughout fruit development in blueberry and bilberry. *DFR* expression was high even in the non-pigmented blueberry fruit flesh, likely associated with proanthocyanidin biosynthesis (Lafferty et al., 2022b), while *F3H1* was less expressed in bilberry fruit skin and flesh compared with the candidate genes and *VcMYBA1*. This result confirmed the expression patterns previously reported from RNA-Seq (Günther et al., 2020), which correlated transcript abundance of the three genes to anthocyanin accumulation over fruit development in blueberry fruit skin and flesh. Analysis of the expression of some flavonoid genes had previously been performed on whole bilberry fruit as well as fruit skin and flesh showing their correlation with anthocyanin accumulation (Jaakola et al., 2002; Lafferty et al., 2022b). Our experiments using qRT-PCR further confirmed the connection between the expression of *CHS1*, *ANS*, *UFGT2* and anthocyanin accumulation in *Vaccinium* fruit. Moreover, the exact same primers were used for both bilberry and blueberry, showing that conserved genes were amplified between the two species.

Phylogenetic analysis showed that CHS1, ANS and UFGT2 predicted protein sequences are highly conserved between blueberry and bilberry, and clustered well with related protein sequences from other plant species, in particular with fruit from the same taxonomic order (Ericales). For all three genes, bilberry and blueberry nucleotide and amino acid sequences showed high sequence similarity (95–99%), which validated the use of the same candidate gene sequences for functional characterisation from both *Vaccinium* species. The active sites of all three genes are highly conserved among anthocyanin-rich fruit species, supporting their relevance in anthocyanin production.

Overexpression of *Vaccinium* R2R3 MYBA-type TFs in *N. benthamiana* leaves induced the expression of endogenous anthocyanin biosynthetic genes which led to anthocyanin production (Karppinen et al., 2021; Lafferty et al., 2022b). Hence, the infiltration of low concentration of *Agrobacterium* carrying *VcMYBA1* in *N. benthamiana* leaves was successfully employed in our experiments to induce a base level of anthocyanin production, which can be elevated through co-infiltration of candidate structural genes.

Transient overexpression in *N. benthamiana* leaves using both the bilberry *VmCHS1* and apple *MdCHS2* did not enhance anthocyanin production above endogenous tobacco CHS that might be induced by VcMYBA, suggesting that CHS is not a rate-limiting step in in this testing system. However, it is well-known that expression of *CHS* is critical for the production of anthocyanins and flavonoids in general. Downregulation/silencing of *CHS* has resulted in abolished or decreased anthocyanin accumulation in many plant species, including tobacco (Wang et al., 2006), kiwifruit (Sun et al., 2014), strawberry (Hoffmann et al., 2006), gentian (Nakatsuka et al., 2008; Ohta et al., 2022) and dahlia (Ohno et al., 2011). Especially, *MdCHS2*-KO transgenic apple lines had no detectable anthocyanins, together with reduced flavonoid accumulation and other changes in plant development (Dare et al., 2013). In our assay, co-infiltration with low amounts of *VcMYBA1* was required to initiate anthocyanin production. *VcMYBA1* has previously been found to control the activation of anthocyanin structural genes, such as *DFR*, *ANS* and *UFGT* (Plunkett et al., 2018; Lafferty et al., 2022a).

In contrast to *VmCHS1*, *VmANS* was shown to significantly increase anthocyanin production by 3 to 3.5 times using *in vivo* expression in *N. benthamiana*. This demonstrated a key role of ANS as a rate-limiting step for anthocyanin accumulation in *N. benthamiana*. ANS had previously been identified as a key enzyme for anthocyanin production in potato tuber (Zhang et al., 2020), *Salvia miltiorrhiza* (Li et al., 2019), apple (Szankowski et al., 2009), *Medicago* (Pang et al., 2007) and cotton (Long et al., 2018). Here, we showed that, of the tested candidate gene combinations, only ANS had a significant effect on anthocyanin production and this is in contrast to a number of previous studies showing that it was the combined expression of ANS with other structural genes, including *UFGT* and *CHS*, that correlated with increased anthocyanin production. For instance, in pink-flowered strawberry (*Fragaria × Potentila*), *FpANS* and two glycosyltransferases (*FpBZ1* and *FpUGT75C1*) were found to be key factors in modulating anthocyanin accumulation in flower petals (Xue et al., 2019). Similarly, upregulation of both *VvANS* and *VvUFGT* in grape were found to correlate with increased anthocyanin accumulation (Yang et al., 2021). In the case of our study, the emphasis on the function of an individual gene, namely *VmANS*, to achieve a significantly higher anthocyanin concentration might be due to the co-expression to a low degree of *VcMYBA1*. Overexpression of bilberry *VmMYBA1* has been shown to induce all endogenous *N. benthamiana* anthocyanin biosynthetic genes (Karppinen et al., 2021). Similarly, the low expression of *VcMYBA1* in our optimised *N. benthamiana* transient assay allowed for the production of anthocyanins *via* the expression of native structural anthocyanin genes, for example, the stabilisation of anthocyanins by *NbUFGT*. Hence, substrates for anthocyanin production were not competed with by other pathways such as the proanthocyanin one. Indeed, when *ANR*, an proanthocyanin structural gene, was silenced in grape and strawberry, expression of *ANS* and anthocyanin accumulation increased (Fischer et al., 2014; Yang et al., 2021). Nevertheless, *ANS* expression was found to associate with anthocyanin accumulation in all aforementioned studies, which further supports the finding that ANS plays a key role in anthocyanin accumulation in combination with MYBA1. Intriguingly, both our qRT-PCR results and previous RNA-Seq data (Lafferty et al., 2022b) showed low, but not total absence of *VcMYBA1* and *VcANS* expression in blueberry flesh despite the tissue not producing anthocyanins and similar observations have been reported for red-skinned but anthocyanin-deficient Muscat Hamburg grape (Xie et al., 2015). As overexpression of *VmANS* was able to elevate anthocyanin production in a low *VcMYBA1* expression background in *N. benthamiana* leaves, it will be insightful to study fruit flesh anthocyanin production in stable ANS- as well as MYBA1- overexpressing blueberry plants to see if flesh colour can be induced. Evidently both genes are good candidates to evaluate as molecular markers for flesh colour in *Vaccinium* spp.

Our infiltration system showed that *VcUFGT2* is important for modulating anthocyanin composition by modifying the sugar moiety of cyanidin, delphinidin and pelargonidin. Therefore, *VcUFGT2* can utilise anthocyanidins as substrate and can be referred to as an anthocyanindin 3*-O* glycosyltransferase. Kubo et al. (2004) suggested that preference for UDP-glucose and UDP-galactose can be ascribed to the last amino acid residue of the PSPG motif, dependent on whether this is a glutamine or a histidine, respectively. Various characterised glucosyltransferases and galactosyltransferases follow this pattern such as residue Q382 of the *Arabidopsis* glucosyltransferase *AtUGT78D2* (Kubo et al., 2007) and residue H375 of the kiwifruit galactosyltransferase *AcF3GT1* (Montefiori et al., 2011). As both VmUFGT2 and VcUFGT2 have a histidine at this position (H376 and H378, respectively), it is likely that they can utilise UDP-galactose as a sugar donor. This is consistent with transient assay results where the production of additional, not naturally occurring galactosylated anthocyanins were observed following overexpression of *VcUFGT2* in *N. benthamiana* leaves and strawberry fruit. Phylogenetic analysis of the UFGT sequences from other plant species showed that most UFGT amino acid sequences from blueberry and bilberry cluster well with other UFGT sequences from the order Ericales, except for VcUFGT3 and VcUFGT5. Interestingly, both the latter two have a glutamine at the conserved last amino acid of the PSPG motif. This suggests that these two enzymes may have a higher affinity for UDP-glucose as their sugar donor, unlike Vc/VmUFGT2. This amino acid residue, however, may not fully determine the sugar donor specificity of a glycosyltransferase as suggested in studies on freesia Fh3GT1, grape VvGT1 and kiwifruit AcF3GT1 (Offen et al., 2006; Montefiori et al., 2011; Sun et al., 2016). For instance, though VvGT1 was shown to accept both glucose and galactose as sugar donor, 200-fold reduced catalytic activity was seen when galactose was used as substrate (Offen et al., 2006). Indeed, the presence of both additional galactosylated and arabinosylated anthocyanins without trade-off in content of glucosylated anthocyanins in strawberry fruit suggested that galactose is not the sole sugar donor accepted by VcUFGT2. Glycosyltransferases can also catalyse the transfer of UDP-sugars to the sugar moiety of anthocyanidin glycosides (GGTs). Such enzymes have previously been characterised in tobacco and kiwifruit (Montefiori et al., 2011; Yonekura‐Sakakibara et al., 2012). Considering our transient assay results and the anthocyanin profile of blueberry, it is unlikely for VcUFGT2 to have GGT activity. Anthocyanidin-3-*O*-glucosides are a major glycoside group in blueberry, although proportions were shown to greatly differ between species with galactosides representing the primary sugar moiety in anthocyanins from Rabbiteye (Günther et al., 2020; Mengist et al., 2020). Hence, it is likely that VcUFGT2 can also accept glucose as sugar donor, although different isoforms, such as VcUFGT3 and VcUFGT5, may also be responsible for glycosylation of glucose. Because the *in vivo* assays used to characterise VcUFGT2 in this study was not suitable for assessing VcUFGT2 affinity to UDP-glucose, future work employing *in vitro* enzyme assays or an *in vivo* system using UFGT-KO strawberry would be needed for this. Regardless, our development of the strawberry transient system overexpressing both *VcMYBA* and *AmF3´5´H* was shown to be a fast and high throughput system useful for *in vivo* qualitative functional test.

A *Vaccinium* fruit transient assay system such as the non-anthocyanic blueberry flesh, would be ideal for verifying the functionality of *Vaccinium* anthocyanin structural genes, particularly for *ANS*. However, this has been proven difficult due to stress-induced anthocyanin biosynthesis when blueberry fruit is injected with *Agrobacterium* during the transient expression process (Günther et al., 2022). Nonetheless, our experimental findings for the function of *VmANS* and especially, *VcUFGT2* in modulating anthocyanin concentrations in heterologous transient expression systems (*N. benthamiana* leaf and strawberry fruit) suggested fundamental key roles for these enzymes in the biosynthesis pathway. Further research on these target enzymes may provide an alternate strategy for the production of specific anthocyanin products. Potentially, in addition to important anthocyanin-related MYB TFs (e.g. *MYBA1*), ANS and UFGT may also be co-targeted to fine-tune high-quality blueberry cultivars with superior anthocyanin profiles.

In conclusion, we have shown that *VmANS* activity is a rate-limiting step within the flavonoid pathway, modulating the anthocyanin concentration *in plantae*. Co-expression with either *VmCHS* or *VcUFGT2* did not further increase anthocyanin content. *VcUFGT2* played a critical role in modulating anthocyanin composition *via* the modification of the sugar moiety. VcUFGT2 also demonstrated affinity for both galactose and arabinose as sugar donor. Our study provided evidence for the key roles of these two final steps in the *Vaccinium* anthocyanin biosynthesis pathway and contributed a new breeding target for novel blueberry cultivars.

## Data availability statement

The datasets presented in this study can be found in online repositories. The names of the repository/repositories and accession number(s) can be found in the article/supplementary material.

## Author contributions

HN performed the experimental work, data analysis and wrote the manuscript. CG cloned *VcUFGT2*, contributed to data analysis and writing of the manuscript. YP cloned and performed functional characterisation of AmF3´5´H. CG and JC performed LTQ LC-MS experiments and data analysis. ES assisted with the cloning and bioinformatic analysis of *VcUFGT2*. BP and AD assisted with cloning of *VmCHS1* and *VmANS*. NA contributed in figure generation and editing. CG conceptualised the study and supervised all aspects with RE and JP. All authors contributed to the article and approved the submitted version.
