# Supplementary material for: Two genes, ANS and UFGT2, from Vaccinium spp. are key steps for modulating anthocyanin production
Source: Front Plant Sci. 2023 Feb 2;14:1082246. doi: 10.3389/fpls.2023.1082246 (PMC9933871; doi:10.3389/fpls.2023.1082246)
Supplement: Supplementary file 1 [file DataSheet_1.pdf]

Research datasets underlying this study can be accessed via the public Mendeley repository:

Guenther, Catrin; Nguyen, Han M (2023), “Research Data for "Two genes, ANS and UFGT2, from Vaccinium spp. are key steps for modulating anthocyanin production ".”, Mendeley Data, V1, doi: 10.17632/bkyvdgfj3c.1

Supplementary data:

Supplementary table 1: Sequences of primers used for qPCR analysis

Gene	Forward primer	Reverse primer
VcMYBA1	5'-GTGGGATGACATGTTGTTCG-3'	5'-CCCACCAACTCCTTGTGT-3'
VmMYBA1	5'-CTCTCCAACCCATCCCAAAC-3'	5'-ACCGTTATCCACCATCATGG-3'
VcCHS1/VmCHS1	5'-AGGTCGAGAAGCCCTTGTTT-3'	5'-GAAAGTCAGGCCCACTTCAC-3'
VcCHS2/VmCHS2	5'-CCTGAAAGAGAACCCAAGTGTG-3'	5'-GCTTTGGTTGCAGCCTCTTT-3'
VcDFR/VmDFR	5'-AATGGCCCGAGTACAATGTC-3'	5'-TGAAACCCCATCCCTATCAA-3'
VcF3H1/VmF3H1	5'-GCTCCCAATGCAACTGTGTA-3'	5'-CCTTGGTCATCTTCCTCCTG-3'
VcANS/VmANS	5'-TCTTCTACGAGGGCAAATGG-3'	5'-GCTCAAAATCTCGACCGTGT-3'
VcUFGT2/VmUFGT2	5'-ACCGCAGTTGCCATAAACTC-3'	5'-TTGAGTTGAACGGACCACAA-3'
VcGAPDH/VmGAPDH	5'-GGTTATCAATGAGTTTGGCA-3'	5'-CAGTCCTTGCTTGATGGACC-3'
VcSAND/VmSAND	5'-AAGCATCTCTTCATCCTGATGA-3'	5'-GATTGTATCTTGGCAGGCAA-3'

Supplementary table 2: Primers used for cloning. Primers were designed for either the sense (forward) or antisense (reverse) of the DNA sequence.

**Primer name**	**Sequence**	**Gene**
5' VmANS + BamHI	5'-GGATCCATGGTGAGTACAATGGT-3'	*VmANS* (forward)
3' VmANS + HindIII	5'-AAGCTTAAGCCCCATTAAGTGC-3'	*VmANS* (reverse)
5' *VmCHS* + BamHI	5'-GGATCCATGGTGACCGTCGA-3'	*VmCHS1* (forward)
3' VmCHS + HindIII	5'-AAGCTTCTAAGTGCACAGACTATG-3'	*VmCHS1* (reverse)
3'VcUFGT2	5'-GGGGACAAGTTTGTACAAAAAAGCAGGCTCCATGTCCAACTTCTCAAAAGACC-3'	*VcUFGT2* (forward)
5' VcUFGT2	5'-GGGGACCACTTTGTACAAGAAAGCTGGGTCTAAATGTTGTACCCTCTCAC-3'	*VcUFGT2* (reverse)
RPH-144	5'-AGGAAGTTCATTTCATTTGGAGAGG-3'	*pGreen* vector
KL106	5'-TTATATGCTCAACACATGAGCGA-3'	*pGreen* vector

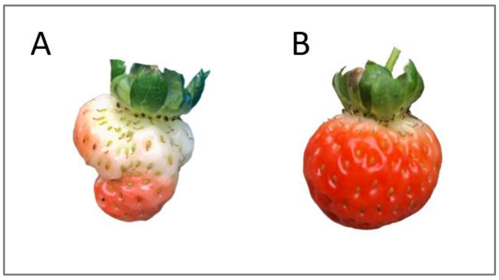

Supplementary figure 1: Strawberry fruit image at initial reddening stage (A) and fully ripe (B) as describe by (Jia et al., 2020)

Method for validation of transgene expression following Agrobacterium-mediated transient transformation in N. benthamiana:

Standard end-point PCR of transformed tissue cDNA was performed on the T100^{TM} Thermal Cycler (Bio-Rad) to confirm the expression of transformed genes (VmANS, VmCHS1 and *VcMYBA1*). All PCR reactions were amplified using the Platinum *Taq* DNA Polymerase (Thermo Fisher Scientific) and other provided components. PCR reaction mixes and conditions are listed in Supplementary table 3.

Supplementary table 3: PCR conditions (a) and reaction mix (b)

a

**Cycles**	**Step**	**Temperature (°C)**	**Time**
1x	Initial denaturation	94	4 min
35x	Denaturation	94	30 sec
	Annealing	57-58*	30 sec
	Extension	72	3 min
1x	Final extension	72	10 min

*Annealing temperature was 58°C for *VmPAL2*, 57°C for *VmCHS1* and *VmANS*

b

**Component**	**25 µL reaction**	**Final concentration**
10x PCR buffer, -Mg	2.5 µL	1x
50 mM $MgCl_2$	0.75 µL	1.5 mM
10 mM dNTP mix	0.5 µL	200 µM
10 ng/µL primers (forward + reverse)	1 µL	0.2 µM each
DNA template	2 µL	Variable
Platinum *Taq* polymerase	0.2 µL	2 U/reaction
Water, nuclease-free	18.05 µL	-

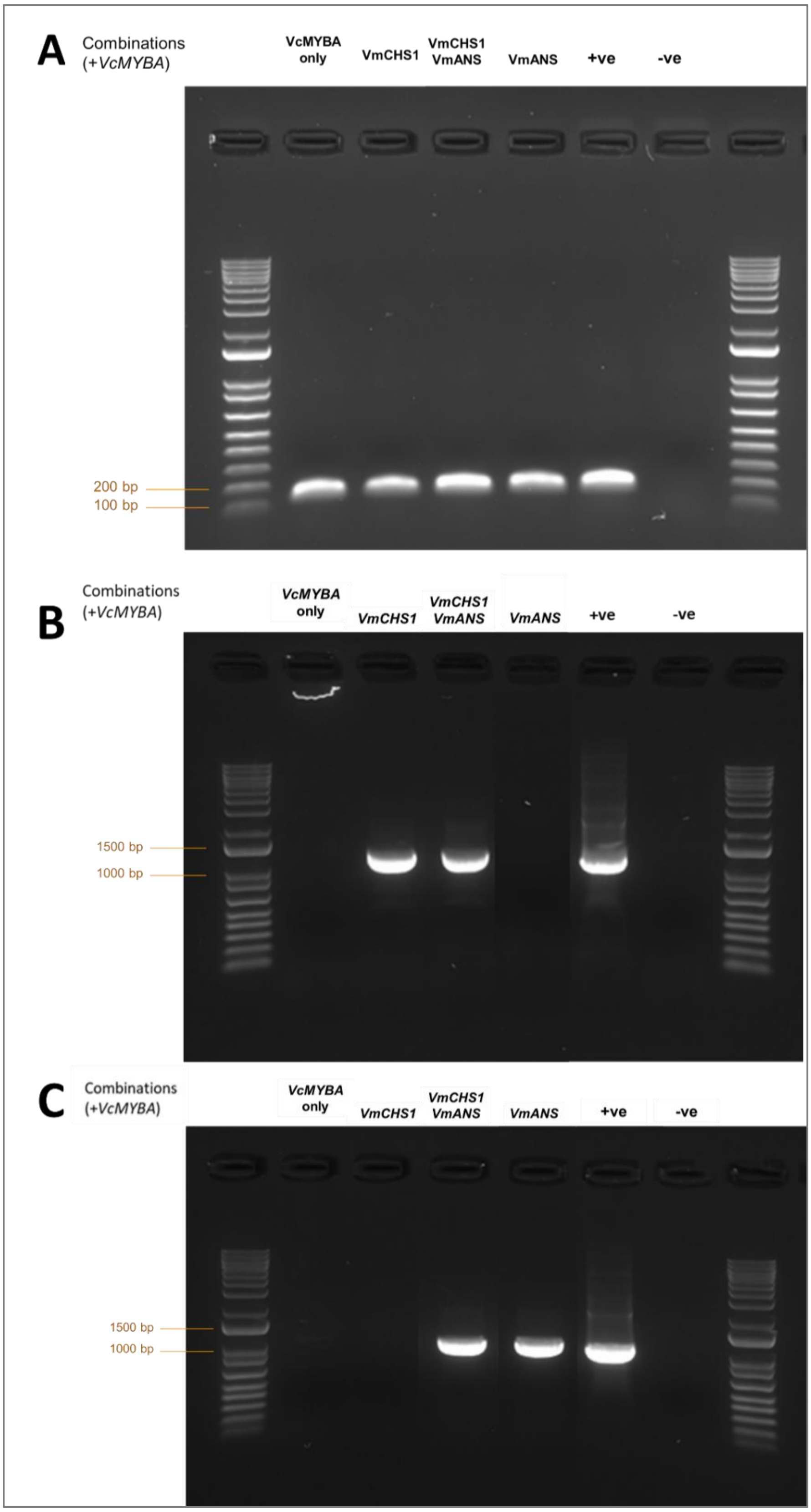

Supplementary figure 2: Electrophoresis gel of endpoint PCR to confirm gene expression of VcMYBA **(A)**, VmCHS1 **(B)**, VmANS **(C)** in *N. benthamiana* leaf.

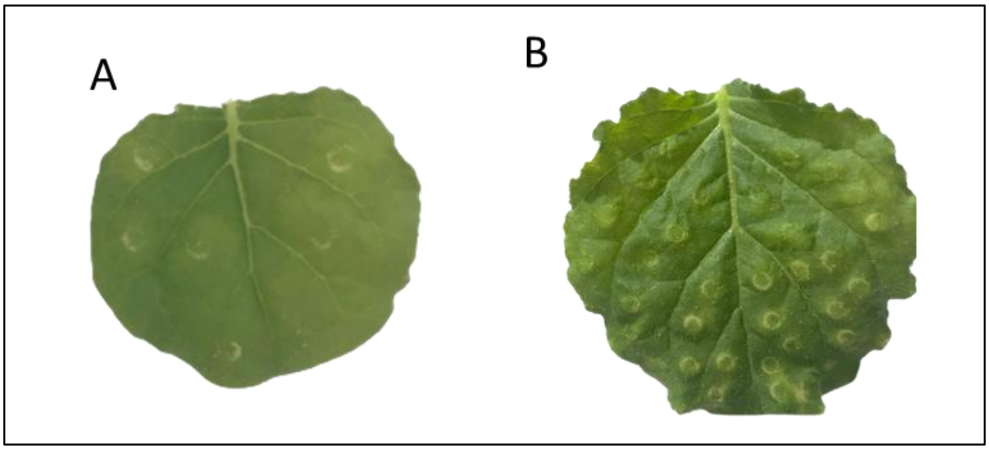

Supplementary figure 3: Image of *N. benthamiana* leaves 5 days after infiltration with *VmCHS1* + *VmANS* + *VcUFGT2* **(A)** and *GUS* **(B)**.

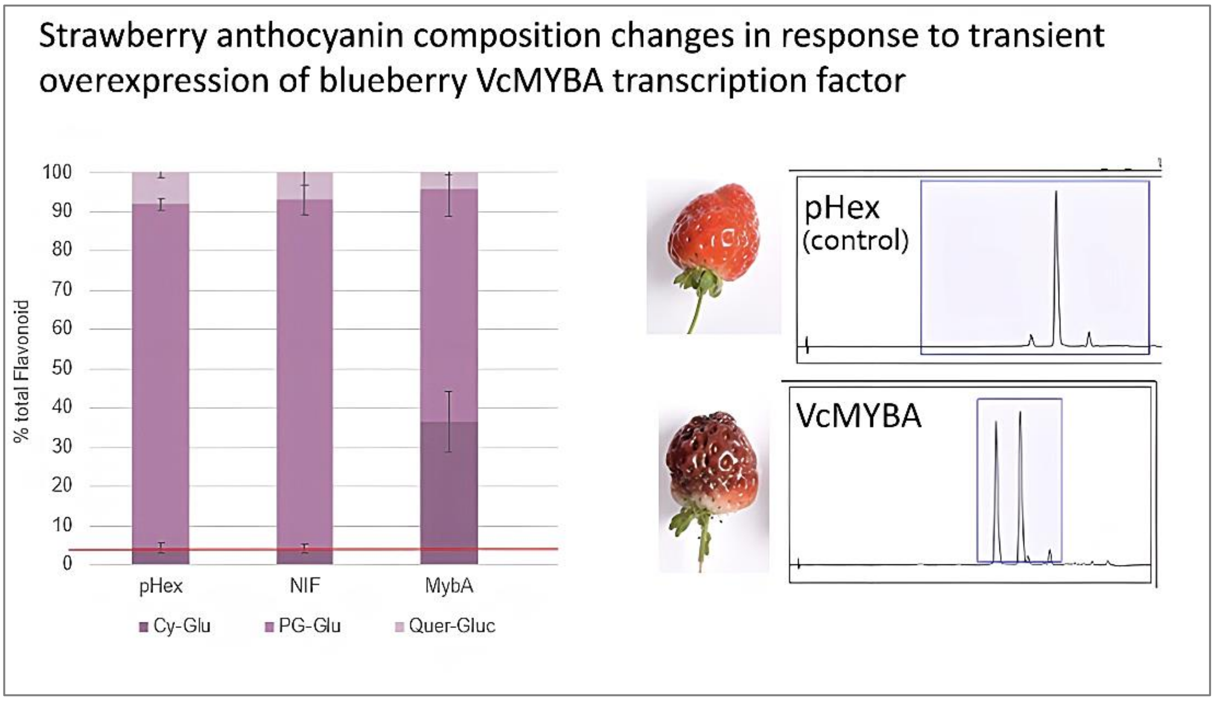

Supplementary figure 4: Strawberry flavonoid composition changes in response to transient overexpression of blueberry *VcMYBA1* transcription factor. Bar graphs show proportional concentration ± standard deviation (N=4). NIF: Non infiltrated control, pHex: *A. tumefaciens* empty vector control, VcMYBA: *Agrobacterium tumefaciens* harbouring *35Spro:VcMYBA1.* Chromatograms showing order of integrated peaks at 520nm. From left to right: cyanidin-3-*O*-glucoside (Cy-Glu); pelargonidin-3-*O*-glucoside (PG-Glu); Quercetin-3-*O*-glucuronide (Quer-Gluc).

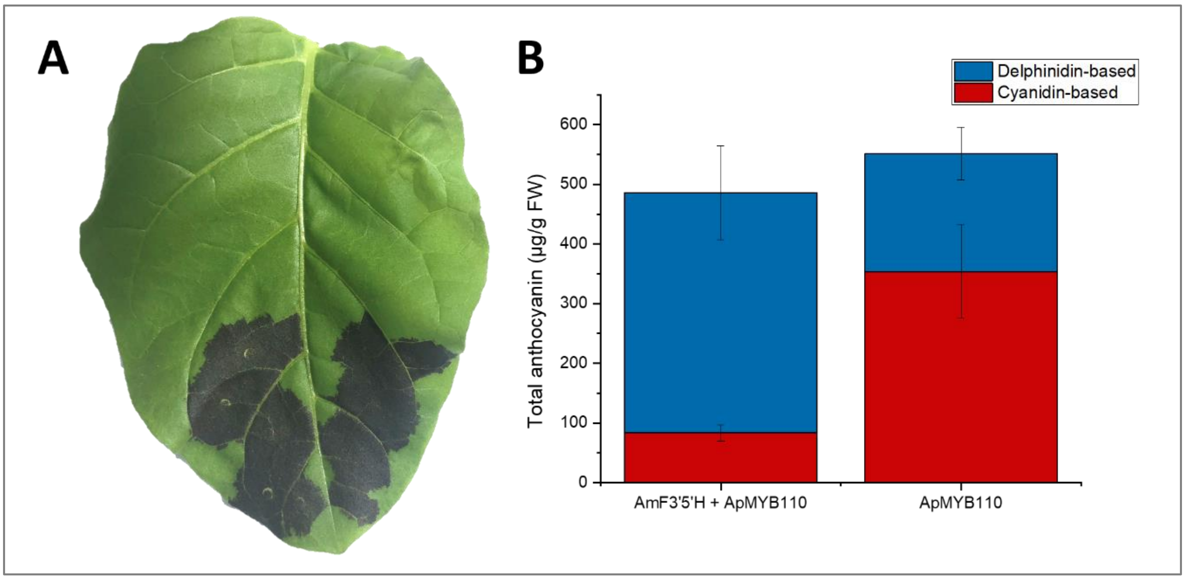

Supplementary figure 5: Transient overexpression of kiwifruit *F3'5'H* co-infiltrated with kiwifruit *MYB110* in *Nicotiana tabacum* leaves. **(A)** Photo of *N. tabacum* leave 7 days after co-infiltration of construct overexpressing *A. melanandra F3´5´H* and construct overexpressing *A. purpurea MYB110*. **(B)** Concentration of cyanidin-based and delphinidin-based anthocyanins measured in the *N. tabacum* leaves 7 days after co-infiltration of *AmF3´5´H*with *ApMYB110* compared to *ApMYB110* alone. The anthocyanin produced by the co-infiltration of *AmF3´5´H*with *ApMYB110* comprised approximately 80% of delphinidin-based anthocyanin, whereas *ApMYB110* alone only induced approximately 30% of delphinidin-based anthocyanin. Data were shown as means ± SEM of three individual experiments.

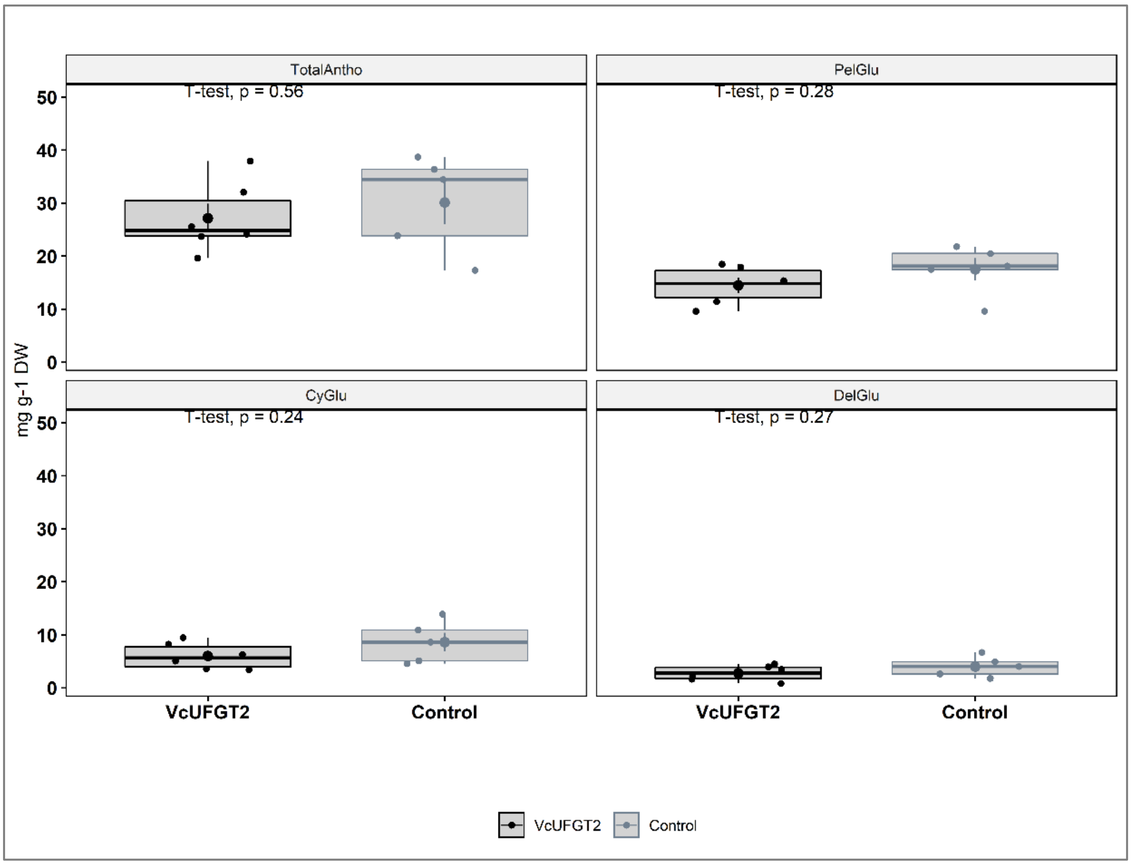

Supplementary figure 6: Box plot visualising mean concentration ± standard deviation (N=6) of total anthocyanin and anthocyanidin-3-O-glucosides of strawberry following strawberry transient transformation 5 days after infiltration. Glu: Glucoside, Pel: Pelargonidin; Cy: Cyanidin; Del: Delphinidin. P-value shows Student's t-test results between *VcUFGT2* and control samples (both in a *VcMYBA1* and *AmF3´5´H*–co-expression background).

Supplementary table 4: Accession number, source database and species of protein sequences used for the phylogenetic analysis of CHS

**Sequence name**	**Accession number**	**Source**	**Species**
Alfalfa_MsCHS2	P30074.1	https://www.ncbi.nlm.nih.gov/protein	*Medicago sativa*
Alfalfa_MsCHS4	P30075.2	https://www.ncbi.nlm.nih.gov/protein	*Medicago sativa*
Apple_MdCHS	ACJ54532.1	https://www.ncbi.nlm.nih.gov/protein	*Malus domestica*
Arabidopsis_AtCHS	AT5G13930	Berardini *et al.*, 2015	*Arabidopsis thaliana*
Azalea_RsCHS	CAC88858.1	https://www.ncbi.nlm.nih.gov/protein	*Rhododendron simsii*
Bilberry_VmCHS1	Vmy03.g8628.t1	Wu *et al.*, 2021	*Vaccinium myrtillus*
Bilberry_VmCHS2	Vmy02.g30348.t1/VmyS15910.g14409.t1*	Wu *et al.*, 2021	*Vaccinium myrtillus*
Blueberry_VcCHS1	maker-VaccDscaff35-augustus-gene-1.21-mRNA-1	Colle *et al.*, 2019	*Vaccinium corymbosum*
Blueberry_VcCHS2	maker-VaccDscaff32-augustus-gene-318.21-mRNA-1	Colle *et al.*, 2019	*Vaccinium corymbosum*
Blueberry_VcCHS3	maker-VaccDscaff2-augustus-gene-67.14	Colle *et al.*, 2019	*Vaccinium corymbosum*
Camellia_CsCHS2	XP_028080516.1	https://www.ncbi.nlm.nih.gov/protein	*Camellia sinensis*
Cassava_MeCHS	XP_021608720.1	https://www.ncbi.nlm.nih.gov/protein	*Manihot esculenta*
CherryPlum_PcCHS	AKV89241.1	https://www.ncbi.nlm.nih.gov/protein	*Prunus cerasifera*
Coffee_CaCHS2	XP_027118978.1	https://www.ncbi.nlm.nih.gov/protein	*Coffea arabica*
CorkOak_QsCHS1	XP_023914191.1	https://www.ncbi.nlm.nih.gov/protein	*Quercus suber*
Cotton_GhCHS1-like	NP_001313780.1	https://www.ncbi.nlm.nih.gov/protein	*Gossypium hirsutum*
Cranberry_VmCHS	Unigene21693_All.p1	Polashock *et al.*,2014	*Vaccinium macrocarpon*
Daisy_GhCHS	CAP20328.1	https://www.ncbi.nlm.nih.gov/protein	*Gerbera hybrid cultivar*
Daisy_GhCHS1	P48390.1	https://www.ncbi.nlm.nih.gov/protein	*Gerbera hybrid cultivar*
Daisy_GhCHS3	P48392.1	https://www.ncbi.nlm.nih.gov/protein	*Gerbera hybrid cultivar*
Durian_DzCHS1-like	XP_022761052.1	https://www.ncbi.nlm.nih.gov/protein	*Durio zibethinus*
Durian_DzCHS2-like	XP_022733123.1	https://www.ncbi.nlm.nih.gov/protein	*Durio zibethinus*
Grape_VvCHS	NP_001267879.1	https://www.ncbi.nlm.nih.gov/protein	*Vitis vinifera*
Hibiscus_HsCHS	XP_039071121.1	https://www.ncbi.nlm.nih.gov/protein	*Hibiscus syriacus*
Kale_BoCHS	QCO90242.1	https://www.ncbi.nlm.nih.gov/protein	*Brassica oleracea* var. *viridis*
Kiwifruit_AcCHS	Acc02004.1	Pilkington *et al.*,2018	*Actinidia chinensis*
Lotus_NnCHS	ADD74168.1	https://www.ncbi.nlm.nih.gov/protein	*Nelumbo nucifera*

Mango_MiCHS1	AIY24987.1	https://www.ncbi.nlm.nih.gov/protein	*Mangifera indica*
Mangosteen_GmCHS	ACM62742.1	https://www.ncbi.nlm.nih.gov/protein	*Garcinia mangostana*
Okra_AeCHS	AGW22222.1	https://www.ncbi.nlm.nih.gov/protein	*Abelmoschus esculentus*
Orange_CsCHS1	XP_006489796.1	https://www.ncbi.nlm.nih.gov/protein	*Citrus sinensis*
Orange_CsCHS2	NP_001306986.1	https://www.ncbi.nlm.nih.gov/protein	*Citrus sinensis*
Peach_PpCHS	AFJ49141.1	https://www.ncbi.nlm.nih.gov/protein	*Prunus persica*
Peach_PpCHS2	XP_007198980.2	https://www.ncbi.nlm.nih.gov/protein	*Prunus persica*
Pear_PcCHS	AGL81345.1	https://www.ncbi.nlm.nih.gov/protein	*Pyrus communis*
Pecan_CiCHS2	QCT85331.1	https://www.ncbi.nlm.nih.gov/protein	*Carya illinoinensis*
Plum_PdCHS	AHZ30595.1	https://www.ncbi.nlm.nih.gov/protein	*Prunus domestica*
Pomegranate_PgCHS	XP_031381600.1	https://www.ncbi.nlm.nih.gov/protein	*Punica granatum*
Rabbiteyeblueberry_VaCHS	BAO58434.1	https://www.ncbi.nlm.nih.gov/protein	*Vaccinium ashei*
Raspberry_RiCHS5	AAM90650.1	https://www.ncbi.nlm.nih.gov/protein	*Rubus idaeus*
Raspberry_RiCHS6	AAM90652.1	https://www.ncbi.nlm.nih.gov/protein	*Rubus idaeus*
Raspberry_RiCHS11	AAM90651.1	https://www.ncbi.nlm.nih.gov/protein	*Rubus idaeus*
Rice_OsCHS1	XP_015618054.1	https://www.ncbi.nlm.nih.gov/protein	*Oryza sativa* Japonica Group
Rice_OsCHS2	XP_015646206.1	https://www.ncbi.nlm.nih.gov/protein	*Oryza sativa* Japonica Group
Strawberry_FaCHS	BAE17124.1	https://www.ncbi.nlm.nih.gov/protein	*Fragaria* x *ananassa*
Tobacco_NbCHS1	QDJ00495.1	https://www.ncbi.nlm.nih.gov/protein	*Nicotiana benthamiana*
Tobacco_NtCHS	NP_001312634.1	https://www.ncbi.nlm.nih.gov/protein	*Nicotiana tabacum*
Tobacco_NtCHS2	ANA78328.1	https://www.ncbi.nlm.nih.gov/protein	*Nicotiana tabacum*
Tobacco_NtCHS3	ANA78329.1	https://www.ncbi.nlm.nih.gov/protein	*Nicotiana tabacum*
Tobacco_NtCHS4	ANA78330.1	https://www.ncbi.nlm.nih.gov/protein	*Nicotiana tabacum*
Tomato_SlCHS1	NP_001234033.2	https://www.ncbi.nlm.nih.gov/protein	*Solanum lycopersicum*
Tomato_SlCHS2	NP_001234036.2	https://www.ncbi.nlm.nih.gov/protein	*Solanum lycopersicum*
ValleyOak_QlCHS1	XP_030974637.1	https://www.ncbi.nlm.nih.gov/protein	*Quercus lobata*
Walnut_JrCHS1	XP_018817256.1	https://www.ncbi.nlm.nih.gov/protein	*Juglans regia*
YoshinoCherry_PyCHS	PQP97034.1	https://www.ncbi.nlm.nih.gov/protein	*Prunus yedoensis* var. *nudiflora*

*consensus sequence between the two sequences

Supplementary table 5: Accession number, source database and species of protein sequences used for the phylogenetic analysis of ANS

Sequence name	Accession number	Source	Species
Apple_MdLDOX	P51091.1	https://www.ncbi.nlm.nih.gov/protein	*Malus domestica*
Arabidopsis_AtANS	AT4G22880.1	Berardini *et al.,* 2015	*Arabidopsis thaliana*
AsianPear_PpANS	ADP09379.1	https://www.ncbi.nlm.nih.gov/protein	*Pyrus pyrifolia*
BeachRose_RrANS	AKT74337.1	https://www.ncbi.nlm.nih.gov/protein	*Rosa rugosa*
Bilberry_VmANS	Vmy12.g1433.t1	Wu *et al.,* 2021	*Vaccinium myrtillus*
BlackGojiBerry_LrANS	AHH55331.1	https://www.ncbi.nlm.nih.gov/protein	*Lycium ruthenicum*
Blueberry_VcANS	maker-VaccDscaff46-augustus-gene-11.23-mRNA-1	Colle *et al.,* 2019	*Vaccinium corymbosum*
Blueberry_VcLDOX	AYC35393.1	https://www.ncbi.nlm.nih.gov/protein	*Vaccinium corymbosum*
Buckwheat_FdANS	AHH30830.1	https://www.ncbi.nlm.nih.gov/protein	*Fagopyrum dibotrys*
Buckwheat_FeANS	ADT63066.1	https://www.ncbi.nlm.nih.gov/protein	*Fagopyrum esculentum*
Cacao_TcANS	ADD51356.1	https://www.ncbi.nlm.nih.gov/protein	*Theobroma cacao*
Camellia_CcANS	AFC37246.1	https://www.ncbi.nlm.nih.gov/protein	*Camellia chekiangoleosa*
Camellia_CsANS	QIM55351.1	https://www.ncbi.nlm.nih.gov/protein	*Camellia sinensis*
CardinalCreeper_IhANS	ACS71531.1	https://www.ncbi.nlm.nih.gov/protein	*Ipomoea horsfalliae*
ChineseWhitePear_PbANS	AJD00703.1	https://www.ncbi.nlm.nih.gov/protein	*Pyrus × bretschneideri*
Crabapple_MhANS	AKQ98194.1	https://www.ncbi.nlm.nih.gov/protein	*Malus hybrid cultivar*
Cranberry_VmANS	CL3153.Contig1_All.p1	Polashock *et al.,* 2014	*Vaccinium macrocarpon*
Eggplant_SmANS	ACJ02088.1	https://www.ncbi.nlm.nih.gov/protein	*Solanum melongena*
Grape_VvANS	ABV82967.1	https://www.ncbi.nlm.nih.gov/protein	*Vitis vinifera*
Grape_VvLDOX	NP_001268147.1	https://www.ncbi.nlm.nih.gov/protein	*Vitis vinifera*
HardyKiwi_AaANS	QPD01602.1	https://www.ncbi.nlm.nih.gov/protein	*Actinidia arguta*
Honeysuckle_LcANS	ALU09330.1	https://www.ncbi.nlm.nih.gov/protein	*Lonicera caerulea*
JapaneseMaple_ApANS	AWN08246.1	https://www.ncbi.nlm.nih.gov/protein	*Acer palmatum*
JapaneseMorningGlory_InANS	BAB71810.1	https://www.ncbi.nlm.nih.gov/protein	*Ipomoea nil*
JapanesePersimmon_DkANS	BAN63164.1	https://www.ncbi.nlm.nih.gov/protein	*Diospyros kaki*

JapanesePlum_PsANS	AEN19292.1	https://www.ncbi.nlm.nih.gov/protein	*Prunus salicina* var. *cordata*
Kiwifruit_AcANS	Acc28876.1	Pilkington *et al.*,2018	*Actinidia chinensis* var. *chinensis*
Kiwifruit_AcLDOX	PSS09778.1	https://www.ncbi.nlm.nih.gov/protein	*Actinidia chinensis* var. *chinensis*
MorningGlory_IpANS	ABW69684.1	https://www.ncbi.nlm.nih.gov/protein	*Ipomoea purpurea*
NoddingLily_LcANS	AGC92011.1	https://www.ncbi.nlm.nih.gov/protein	*Lilium cernuum*
Notoginseng_PnANS	QKV26469.1	https://www.ncbi.nlm.nih.gov/protein	*Panax notoginseng*
Peach_PpANS	AJA79070.1	https://www.ncbi.nlm.nih.gov/protein	*Prunus persica*
Pear_PcANS	PCP027029	https://www.ncbi.nlm.nih.gov/protein	*Pyrus communis*
Plum_PdANS	AHZ30597.1	https://www.ncbi.nlm.nih.gov/protein	*Prunus domestica*
Potato_StANS1	AEJ90548.1	https://www.ncbi.nlm.nih.gov/protein	*Solanum tuberosum*
Potato_StANS2	NP_001274859.1	https://www.ncbi.nlm.nih.gov/protein	*Solanum tuberosum*
Raspberry_RiANS	Ro05_G14884	VanBuren *et al.,* 2018	*Rubus occidentalis*
RedRaspBerry_RiANS	AXY97021.1	https://www.ncbi.nlm.nih.gov/protein	*Rubus idaeus*
Sainfoin_OvANS	AEF14411.1	https://www.ncbi.nlm.nih.gov/protein	*Onobrychis viciifolia*
SiamTulip_CaANS1	QPZ56416.1	https://www.ncbi.nlm.nih.gov/protein	*Curcuma alismatifolia*
SourCherry_PcANS	AJO67980.1	https://www.ncbi.nlm.nih.gov/protein	*Prunus cerasus*
Soybean_GmANS1	NP_001239794	https://www.ncbi.nlm.nih.gov/protein	*Glycine max*
Soybean_GmANS2	AAR26526.1	https://www.ncbi.nlm.nih.gov/protein	*Glycine max*
Strawberry_FaANS	FvH4_5g01170.1	Edger *et al.*, 2018	*Fragaria vesca*
SweetCherry_PaANS	AEO79983.1	https://www.ncbi.nlm.nih.gov/protein	*Prunus avium*
SweetPotato_IbANS	BAA75305.1	https://www.ncbi.nlm.nih.gov/protein	*Ipomoea batatas*
Tobacco_NtANS1	AWL24853.1	https://www.ncbi.nlm.nih.gov/protein	*Nicotiana tabacum*
Tobacco_NtANS2	AFM52335.1	https://www.ncbi.nlm.nih.gov/protein	*Nicotiana tabacum*
Tobacco_NtLDOX	NP_001312972.1	https://www.ncbi.nlm.nih.gov/protein	*Nicotiana tabacum*
Tomato_SlANS	Solyc08g080040.4.1	Tomato Genome Consortium, 2012	*Solanum lycopersicum*
TurnipRape_BrANS1	ABY89681.1	https://www.ncbi.nlm.nih.gov/protein	*Brassica rapa* subsp. *oleifera*

Supplementary table 5: Accession number, source database and species of protein sequences used for the phylogenetic analysis of UFGT

**Sequence name**	**Accession number**	**Source**	**Species**
AcGaT	BAD06514.1	https://www.ncbi.nlm.nih.gov/protein	*Aralia cordata*
Almond_PdUFGT2-like	XP_034203307.1	https://www.ncbi.nlm.nih.gov/protein	*Prunus dulcis*
AmurGrape_VaUFGT	ACI15395.1	https://www.ncbi.nlm.nih.gov/protein	*Vitis amurensis*
Apple_MdUFGT1	AAD26203.1	https://www.ncbi.nlm.nih.gov/protein	*Malus domestica*
Apple_MdUFGT2	XP_008357063.1	https://www.ncbi.nlm.nih.gov/protein	*Malus domestica*
Arabidopsis_AtUGT78D2	NP_197207.1	https://www.ncbi.nlm.nih.gov/protein	*Arabidopsis thaliana*
AsUGT	KAG7609479.1	https://www.ncbi.nlm.nih.gov/protein	*Arabidopsis suecica*
Bilberry_VmUFGT1	Vmy10.g10923.t1	Wu *et al.,* 2021	*Vaccinium myrtillus* L
Bilberry_VmUFGT2	Vmy04g29351.t1	Wu *et al.,* 2021	*Vaccinium myrtillus* L
Bilberry_VmUFGT3	VmyS8874.g18298.t1	Wu *et al.,* 2021	*Vaccinium myrtillus* L
Bilberry_VmUFGT4	Vmy09.g23640.t1	Wu *et al.,* 2021	*Vaccinium myrtillus* L
Blueberry_VcUFGT1	maker-VaccDscaff28-augustus-gene-290.34	Colle *et al.,* 2019	*Vaccinium corymbosum*
Blueberry_VcUFGT2	maker-VaccDscaff6-augustus-gene-420.36	Colle *et al.,* 2019	*Vaccinium corymbosum*
Blueberry_VcUFGT3	aker-VaccDscaff37-augustus-gene-300.29	Colle *et al.,* 2019	*Vaccinium corymbosum*
Blueberry_VcUFGT4	maker-VaccDscaff19-snap-gene-64.36	Colle *et al.,* 2019	*Vaccinium corymbosum*
Blueberry_VcUFGT5	maker-VaccDscaff34-augustus-gene-294.29	Colle *et al.,* 2019	*Vaccinium corymbosum*
BogBilberry_VuUFGT	AKE92926.1	https://www.ncbi.nlm.nih.gov/protein	*Vaccinium uliginosum*
Cacao_TcUFGT2	XP_017977055.1	https://www.ncbi.nlm.nih.gov/protein	*Theobroma cacao*
Camellia_CnUFGT15	QWM97336.1	https://www.ncbi.nlm.nih.gov/protein	*Camellia nitidissima*
Carrot_DcUCGalT1	AKI23632.1	https://www.ncbi.nlm.nih.gov/protein	*Daucus carota*
CcUGT	OMO98379.1	https://www.ncbi.nlm.nih.gov/protein	*Corchorus capsularis*
Celery_AgUCGalT1	AXU98426.1	https://www.ncbi.nlm.nih.gov/protein	*Apium graveolens*
CherryPlum_PcUFGT	AKV89253.1	https://www.ncbi.nlm.nih.gov/protein	*Prunus cerasifera*
ChilliPepper_CaUFGT	KAF3667410.1	https://www.ncbi.nlm.nih.gov/protein	*Capsicum annuum*
ChinaRose_RcUFGT2	XP_024179381.1	https://www.ncbi.nlm.nih.gov/protein	*Rosa chinensis*
ChineseBayberry_MrUFGT2	KAB1205527.1	https://www.ncbi.nlm.nih.gov/protein	*Morella rubra*

ChinesePlum_PsUFGT	AMD39598.1	https://www.ncbi.nlm.nih.gov/protein	*Prunus salicina*
ChineseWhitePear_PbUFGT	AGZ15303.1	https://www.ncbi.nlm.nih.gov/protein	*Pyrus* x *bretschneideri*
Corn_ZmF3GT	P16165.1	https://www.ncbi.nlm.nih.gov/protein	*Zea mays*
Cotton_GaUFGT2	KHG23412.1	https://www.ncbi.nlm.nih.gov/protein	*Gossypium arboreum*
Cottonwood_PtUFGT7	XP_006376354.2	https://www.ncbi.nlm.nih.gov/protein	*Populus trichocarpa*
Durian_DzUFGT2-like	XP_022746913.1	https://www.ncbi.nlm.nih.gov/protein	*Durio zibethinus*
Eg3GT	BAF49284.1	https://www.ncbi.nlm.nih.gov/protein	*Eustoma grandiflorum*
Eggplant_SmUFGT	Q43641.1	https://www.ncbi.nlm.nih.gov/protein	*Solanum melongena*
EsUGT78D2	XP_006400242.1	https://www.ncbi.nlm.nih.gov/protein	*Eutrema salsugineum*
FoxGrape_VlUFGT	ABR24135.1	https://www.ncbi.nlm.nih.gov/protein	*Vitis labrusca*
GaUFGT2-like	KAA3457312.1	https://www.ncbi.nlm.nih.gov/protein	*Gossypium australe*
Ginseng_PgUGT14	AIE12487.1	https://www.ncbi.nlm.nih.gov/protein	*Panax ginseng*
Grape_VvUFGT	NP_001384786.1	https://www.ncbi.nlm.nih.gov/protein	*Vitis vinifera*
HabaneroPepper_CcUFGT	PHU05603.1	https://www.ncbi.nlm.nih.gov/protein	*Capsicum chinense*
Hibiscus_HsUFGT-like	XP_039067583.1	https://www.ncbi.nlm.nih.gov/protein	*Hibiscus syriacus*
JapaneseMorningGlory_InUFGT	BAR88267.1	https://www.ncbi.nlm.nih.gov/protein	*Ipomoea nil*
KakiPersimmon_DkF3GT1	BAI40148.1	https://www.ncbi.nlm.nih.gov/protein	*Diospyros kaki*
Kiwifruit_AcF3GT1	ADC34700.1	https://www.ncbi.nlm.nih.gov/protein	*Actinidia chinensis*
Kiwifruit_ArUFGT	GFZ06465.1	https://www.ncbi.nlm.nih.gov/protein	*Actinidia rufa*
Le3GT-A	BAF49310.1	https://www.ncbi.nlm.nih.gov/protein	*Lobelia erinus*
Le3GT-B	BAF49311.1	https://www.ncbi.nlm.nih.gov/protein	*Lobelia erinus*
MexicanCotton_GhUFGT-like	XP_016740936.2	https://www.ncbi.nlm.nih.gov/protein	*Gossypium hirsutum*
MorningGlory_IpUFGT	BAR88272.1	https://www.ncbi.nlm.nih.gov/protein	*Ipomoea purpurea*
NaltaJute_CoUGT	OMO62224.1	https://www.ncbi.nlm.nih.gov/protein	*Corchorus olitorius*
NbUGT	UHH90493.1	https://www.ncbi.nlm.nih.gov/protein	*Nicotiana benthamiana*
Notoginseng_PnUFGT	QKV26470.1	https://www.ncbi.nlm.nih.gov/protein	*Panax notoginseng*
OkinawanSpinach_GbUFGT1a	BAP47702.1	https://www.ncbi.nlm.nih.gov/protein	*Gynura bicolor*
PdUFGT	AQZ26785.1	https://www.ncbi.nlm.nih.gov/protein	*Paeonia delavayi*
Peach_PpUFGT	AFP90753.1	https://www.ncbi.nlm.nih.gov/protein	*Prunus persica*

Peach_PpUFGT2	XP_007217952.1	https://www.ncbi.nlm.nih.gov/protein	*Prunus persica*
Pear_PcUFGT	AGL81353.1	https://www.ncbi.nlm.nih.gov/protein	*Pyrus communis*
Petunia_PhF3GalTase	BAA89008.1	https://www.ncbi.nlm.nih.gov/protein	*Petunia* x *hybrida*
Potato_StUFGT	AKM76367.1	https://www.ncbi.nlm.nih.gov/protein	*Solanum tuberosum*
Ps3GT	QNU13183.1	https://www.ncbi.nlm.nih.gov/protein	*Paeonia suffruticosa*
RabbiteyeBlueberry_VaUFGT	BAO58430.1	https://www.ncbi.nlm.nih.gov/protein	*Vaccinium ashei*
Raspberry_RiUGT	AWT04749.1	https://www.ncbi.nlm.nih.gov/protein	*Rubus idaeus*
RiverbankGrape_VrUFGT	XP_034710694.1	https://www.ncbi.nlm.nih.gov/protein	*Vitis riparia*
Rose_RhUFGT	BAF80946.1	https://www.ncbi.nlm.nih.gov/protein	*Rosa hybrid cultivar*
Stock_MiUFGT	BBM96381.1	https://www.ncbi.nlm.nih.gov/protein	*Matthiola incana*
Strawberry_FaGT1	AAU09442.1	https://www.ncbi.nlm.nih.gov/protein	*Fragaria* x *ananassa*
SweetCherry_PaUFGT	AJO67971.1	https://www.ncbi.nlm.nih.gov/protein	*Prunus avium*
SweetPotato_IbUFGT	AEN25473.1	https://www.ncbi.nlm.nih.gov/protein	*Ipomoea batatas*
Tomato_SlUFGT	XP_004249447.1	https://www.ncbi.nlm.nih.gov/protein	*Solanum lycopersicum*
WildStrawberry_FvUGT1	AJW28718.1	https://www.ncbi.nlm.nih.gov/protein	*Fragaria vesca* subsp. *vesca*
